# EffectorK, a comprehensive resource to mine for *Ralstonia, Xanthomonas,* and other published effector interactors in the *Arabidopsis* proteome

**DOI:** 10.1111/mpp.12965

**Published:** 2020-08-15

**Authors:** Manuel González‐Fuente, Sébastien Carrère, Dario Monachello, Benjamin G. Marsella, Anne‐Claire Cazalé, Claudine Zischek, Raka M. Mitra, Nathalie Rezé, Ludovic Cottret, M. Shahid Mukhtar, Claire Lurin, Laurent D. Noël, Nemo Peeters

**Affiliations:** ^1^ Laboratoire des Interactions Plantes Micro‐organismes, INRAE CNRS Université de Toulouse Castanet‐Tolosan France; ^2^ Institut des Sciences des Plantes de Paris Saclay UEVE INRAE CNRS Université Paris Sud Université Paris‐Saclay Gif‐sur‐Yvette France; ^3^ Université de Paris Gif‐sur‐Yvette France; ^4^ Department of Biology University of Alabama at Birmingham Birmingham AL USA; ^5^ Department of Biology Carleton College Northfield MN USA

**Keywords:** database, effectors, interactomics, network, Ralstonia, Xanthomonas

## Abstract

Pathogens deploy effector proteins that interact with host proteins to manipulate the host physiology to the pathogen's own benefit. However, effectors can also be recognized by host immune proteins, leading to the activation of defence responses. Effectors are thus essential components in determining the outcome of plant–pathogen interactions. Despite major efforts to decipher effector functions, our current knowledge on effector biology is scattered and often limited. In this study, we conducted two systematic large‐scale yeast two‐hybrid screenings to detect interactions between *Arabidopsis thaliana* proteins and effectors from two vascular bacterial pathogens: *Ralstonia pseudosolanacearum* and *Xanthomonas campestris*. We then constructed an interactomic network focused on *Arabidopsis* and effector proteins from a wide variety of bacterial, oomycete, fungal, and invertebrate pathogens. This network contains our experimental data and protein–protein interactions from 2,035 peer‐reviewed publications (48,200 *Arabidopsis*–*Arabidopsis* and 1,300 *Arabidopsis–*effector protein interactions). Our results show that effectors from different species interact with both common and specific *Arabidopsis* interactors, suggesting dual roles as modulators of generic and adaptive host processes. Network analyses revealed that effector interactors, particularly “effector hubs” and bacterial core effector interactors, occupy important positions for network organization, as shown by their larger number of protein interactions and centrality. These interactomic data were incorporated in EffectorK, a new graph‐oriented knowledge database that allows users to navigate the network, search for homology, or find possible paths between host and/or effector proteins. EffectorK is available at www.effectork.org and allows users to submit their own interactomic data.

## Graphical abstract

EffectorK (www.effectorK.org) is an open database compiling newly generated and literature‐curated interactomic data from various *Arabidopsis* pathogen effectors.

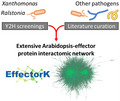

## 1 INTRODUCTION

Plants are continuously confronted with a wide variety of pathogens, including bacteria, oomycetes, fungi, nematodes, and insects. To prevent their proliferation, plants have evolved a complex multilayered immune system (Jones and Dangl, 2006). Plants are able to recognize highly conserved pathogen‐associated molecular patterns (PAMPs) through pattern‐recognition receptors triggering induced defence responses collectively known as PAMP‐triggered immunity (PTI) (Zipfel, 2014). These responses are usually enough to prevent most potential invaders; however, some pathogens secrete effector proteins to subvert the defence responses and alter diverse cellular processes to ease their proliferation (Ma *et al*., 2018). Plants, moreover, have evolved several intracellular nucleotide‐binding site‐leucine‐rich repeat (NBS‐LRR) receptors recognizing these effectors and activating potent defence responses collectively known as effector‐triggered immunity (ETI) (Cui *et al*., 2015).

Although the interactors and molecular functions of some effectors have been characterized (Büttner, 2016; Giron *et al*., 2016; Sharpee and Dean, 2016; Vieira and Gleason, 2019), for most effectors they are still unknown. The main factors complicating the large‐scale identification and characterization of effector–host protein interactions are the wide diversity of pathosystems, the difficulty in identifying bona fide effector genes, the collective contribution of effector proteins, the complexity of the host responses, and the lack of robust high‐throughput techniques. For the model species *Arabidopsis thaliana* (Ath), to our knowledge, there are only two studies in which systematic effector–host protein interactions at the effectome‐scale have been identified (Mukhtar *et al*., 2011; Weßling *et al*., 2014). In these studies plant interactors of effector proteins from *Pseudomonas syringae* (Psy, bacterium), *Hyaloperonospora arabidopsidis* (Hpa, oomycete), and *Glovinomyces orontii* (Gor, fungus) were identified by yeast two‐hybrid (Y2H) assays. They reported that the effectors of these species converged onto a limited set of Ath proteins. These studies also demonstrated that many effector interactors are important for plant immunity and showed that their importance correlates with the level of effector convergence.

Bacterial wilt, caused by *Ralstonia pseudosolanacearum* (*Ralstonia solanacearum* phylotype I, Rps), and black rot, caused by *Xanthomonas campestris* pv. *campestris* (Xcc), are listed among the top 10 scientifically and economically important plant bacterial diseases (Mansfield *et al*., 2012). Both Rps and Xcc are xylem‐colonizing bacteria able to infect the model plant Ath (Deslandes *et al*., 1998; Buell, 2002). They both rely on their type III secretion system for full virulence (Arlat *et al*., 1991, 1992). This “molecular syringe” allows the pathogen to deliver type III effector proteins (T3Es) directly into the host cell in order to promote disease. The roles of several of their T3Es have been characterized (White *et al*., 2009; Coll and Valls, 2013), but most knowledge on T3E functions comes from the study of Psy, which resides on leaf surfaces and in the leaf apoplast (Lindeberg *et al*., 2012; Büttner, 2016). Focusing mainly on a few species offers a partial view of effector biology. It is therefore crucial to expand our studies to other species to grasp the existing diversity of effector proteins and pathogen lifestyles.

To obtain a deeper understanding of the global Ath–effector protein interactome, we conducted three systematic large‐scale screenings with T3Es from Rps and Xcc, the first vascular pathogens screened in this manner. Additionally, we conducted an extensive literature survey to gather published Ath interactors of effector proteins from pathogens from four different kingdoms of life: Bacteria, Chromista, Fungi, and Animalia. Combining all these data allowed us to identify 100 new “effector hubs” (i.e., Ath proteins interacting with two or more effectors). Together with Ath–Ath protein interactions retrieved from public databases, we generated an Ath–effector protein network that captures the wide diversity of Ath pathogens. This network allowed us to detect general trends of effector interference with the host proteome. We have created a publicly available interactive knowledge database called EffectorK (for Effector Knowledge) that allows users to access and augment this network.

## 2 RESULTS

### 2.1 Systematic identification of *Arabidopsis* interactors of *R. pseudosolanacearum* and *X. campestris* effectors

**FIGURE 1:**
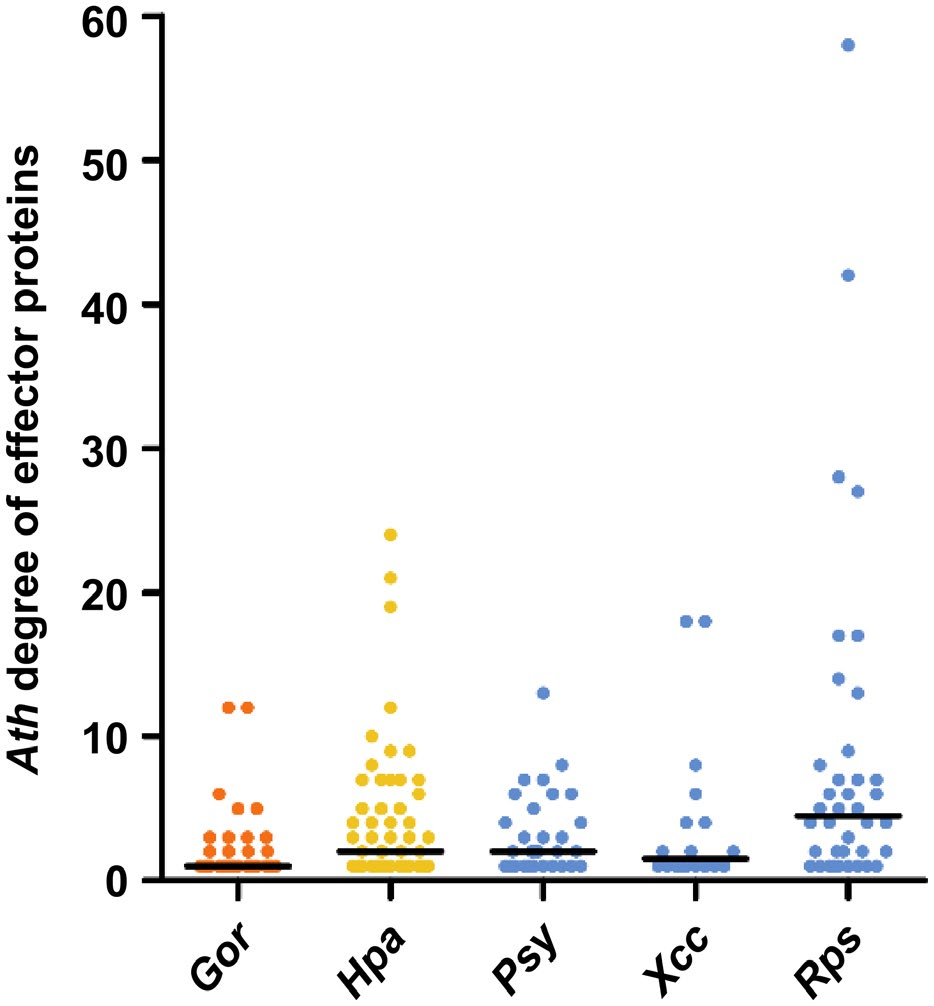
*A* *rabidopsis thaliana* (Ath) degree of effector proteins from *Glovinomyces orontii* (Gor), *Hyaloperonospora arabidopsidis* (Hpa), *Pseudomonas syringae* (Psy), *Xanthomonas* *campestris* pv. *campestris* (Xcc), and *Ralstonia pseudosolanacearum* (Rps). Comparison of the Ath degree (i.e., number of Ath interactors per effector) of effector proteins from Gor, Hpa, Psy, Xcc, and Rps found in the 8,000‐Ath‐cDNA collection (8K space). Horizontal black bars represent the median. Colours represent the kingdom (orange: Fungi, yellow: Chromista, and blue: Bacteria)

Three Y2H screenings were performed to identify Ath interactors of Rps and Xcc effector proteins. In a first screening, we identified 42 Ath interactors for 21 out of 56 T3Es from Rps strain GMI100 screened against a library of more than 8,000 full‐length Ath cDNAs (8K space). The choice of the 56 Rps T3Es was guided by the available clones at the time of screening. In the second and third screenings, we identified 176 Ath interactors for 32 out of 48 T3Es from Rps strain GMI1000 and 52 Ath interactors for 18 out of 25 T3Es from Xcc strain 8,004 screened against an extended version of the previous library containing more than 12,000 Ath full‐length cDNAs (12K space) (Figure S1 and Table S1). Here the choice of Rps T3Es was constrained by a pool maximum imposed by the screening method (see Materials and Methods). T3Es were picked according to their highest degree of conservation within the species complex (Peeters *et al*., 2013). On average, 10.7 and 5.7 Ath interactors were found per Rps and Xcc T3E. These Ath cDNA libraries had been previously used to test interactions with 57 and 32 effector proteins from Hpa and Psy, respectively, (8K space) and 46 effector proteins from Gor (12K space) (Mukhtar *et al*., 2011; Weßling *et al*., 2014). The subset of interactions of effectors from Rps, Xcc, and Gor in the 8K space was used to compare with previously published Hpa and Psy data (Figure 1). In general, Rps effectors interacted on average with more Ath proteins than the other screened species; however, this difference is only statistically significant when compared to Gor effectors (one‐tailed Wilcoxon signed‐rank test *p* < .001). These data show that effector proteins from these five different species, on average, tend to interact with a similar number of Ath proteins regardless of kingdom, life style, or effectome size.

### 2.2 Effectors converge onto a limited set of *Arabidopsis* proteins

We compared the Rps and Xcc effector interactors identified in our screenings with the interactors previously identified for Hpa, Psy, and Gor effector proteins (Mukhtar *et al*., 2011; Weßling *et al*., 2014). To avoid bias related to the size of the screened library, we considered only the subset of effector interactors present in the 8K space (Figure S2). At the kingdom level, Bacteria was the kingdom with the highest number of kingdom‐specific interactors, with 158 exclusive interactors out of a total of 217 interactors (72.8%), followed by Chromista, with 31 out of 117 (51.7%), and Fungi, with 16 out of 45 (35.6%). In total, 235 out of 299 effector interactors (78.6%) were kingdom‐specific. At the species level, when comparing all five pathogens, the percentage of species‐specific interactors was 58.9% for Psy, 58.7% for Rps, 51.7% for Hpa, 48.8% for Xcc, and 35.6% for Gor. The total number of species‐specific effector interactors was 221 out of 299 (73.9%). These data show that most effector interactors are kingdom‐ and species‐specific.

**FIGURE 2:**
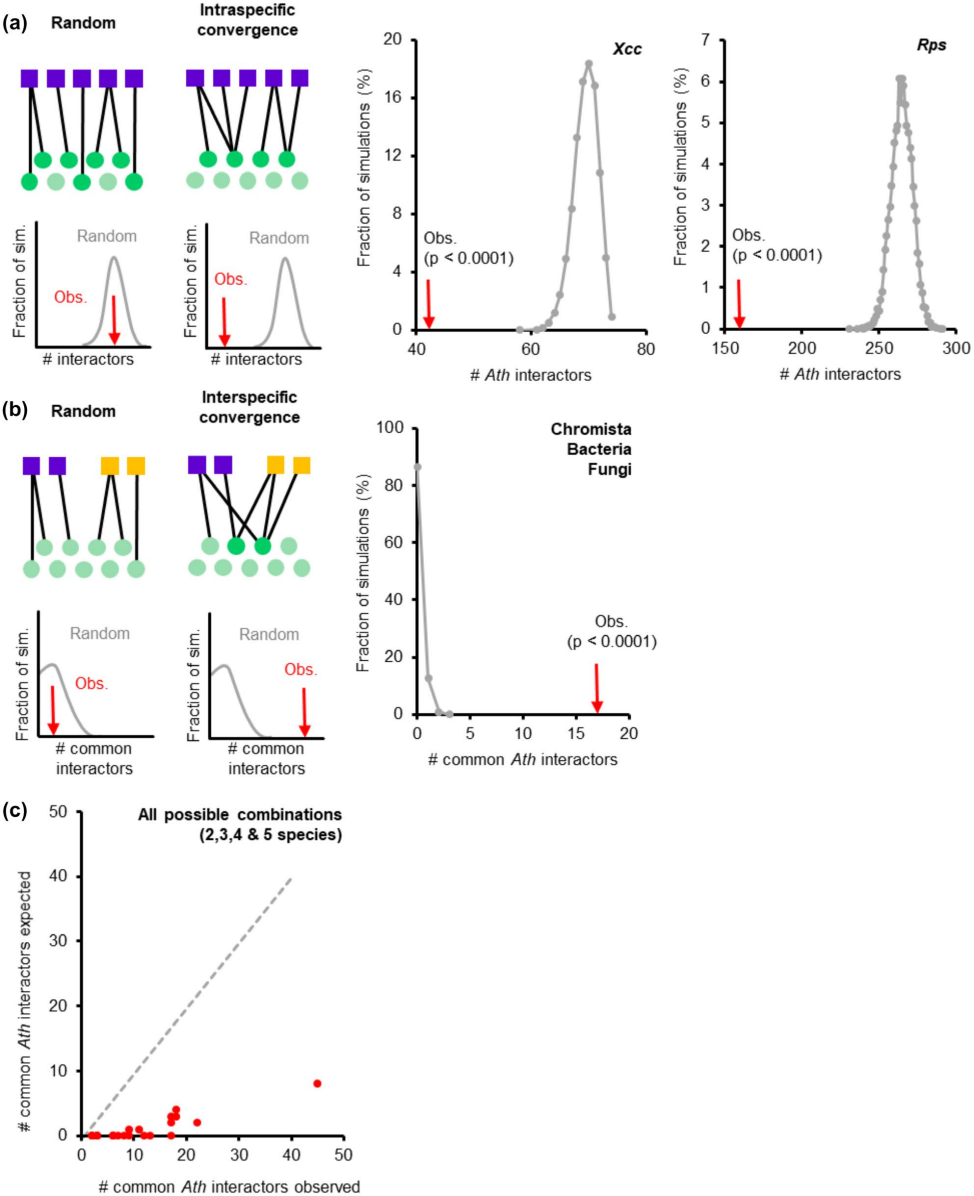
Effectors converge intra‐ and interspecifically onto a common set of *Arabidopsis thaliana* (Ath) proteins. (a) Left: random and intraspecific convergent interactions of effectors (purple squares) with Ath proteins (green circles) can be distinguished by random network rewiring and simulation. Adapted from Weßling *et al*. (2014). Middle and right: number of Ath interactors in the 8K space of effectors from *Xanthomonas* *campestris* pv. *campestris* (Xcc) strain 8,004 and *Ralstonia pseudosolanacearum* (Rps) strain GMI1000 found in 10,000 degree‐preserving simulations (grey) versus the observed number (red arrow). (b) Left: random and interspecific convergent interactions of effectors from different species (purple and orange squares) with Ath proteins (green circles) can be distinguished by random network rewiring and simulation. Right: number of common Ath interactors in the 8K space of effectors from Chromista, Bacteria, and Fungi found in 10,000 simulations (grey) versus the observed number (red arrow). (c) Scatterplot of observed versus simulated number of common Ath interactors between all binary, ternary, quaternary, and quinary combinations of species. *x* = *y* regression is represented with a dashed grey line

To evaluate whether Rps and Xcc effectors interact randomly or converge onto a common set of Ath proteins we performed simulations rewiring effector–Ath protein interactions within the 8K space. In these simulations, each effector was assigned randomly as many Ath proteins as it had interacted with in our screenings. Then, the number of interactors found on all simulations was plotted and compared with the experimental data (Figure 2a). The number of effector interactors observed in our screenings was significantly lower than the numbers obtained in the random simulations for both Rps and Xcc. Similar results had been reported for effectors from Hpa, Psy, and Gor (Mukhtar *et al*., 2011; Weßling *et al*., 2014). This shows that, similarly to other species, both Rps and Xcc effectors also interact with a common subset of Ath proteins (i.e., intraspecific convergence).

These random rewiring simulations also allowed us to determine whether effectors from different species interact randomly or convergently with Ath proteins. For this, the number of common interactors of effectors from different species was compared with the experimental data (Figure 2b). When comparing all three kingdoms, the number of common interactors observed was significantly higher than expected by random rewiring. We then analysed all possible binary, ternary, quaternary, and quinary combinations of species and in all cases the number of common interactors observed was higher than expected randomly (Figure 2c). These differences were all statistically significant except for the common interactors of effectors from Psy and Xcc (*p* = .058; Figure S3). This could indicate that these two species are the most different in terms of effector targeting. However, considering that Psy and Xcc are precisely the two species with the lowest number of effectors for which interactors have been identified (Psy: 32 and Xcc: 18 effector proteins), it is likely that the high *p* value is caused by the limited sample size. This shows that effectors from all these five species interact with a common subset of Ath proteins (i.e., interspecific convergence).

Altogether, our data indicate that Rps and Xcc effectors converge both intra‐ and interspecifically onto a set of limited Ath proteins, behaving similarly to effectors from other previously screened pathogen species. This suggests the existence of a convergent set of effector interactors common to evolutionarily distant pathogens that might have a predominant role in the general modulation of the host responses.

### 2.3 Manual curation of the literature to compile *Arabidopsis–*effector protein interactions

In order to gather more knowledge on Ath–effector protein interactions, we conducted an extensive literature search compiling data from a wider spectrum of bacterial, fungal, oomycete, and invertebrate effector proteins. We only considered published direct protein–protein interactions that had been confirmed by classic techniques such as Y2H, co‐immunoprecipitation, pull‐down, protein‐fragment complementation, fluorescence resonance energy transfer, or mass spectrometry. We compiled 287 interactions found in 80 peer‐reviewed publications involving 218 Ath proteins and 72 effectors from 22 pathogen species (Table S2). Among these 22 pathogens, there were nine bacterial species, mostly proteobacteria but also a phytoplasma species; eight invertebrate species, including both nematodes and insects; four oomycete, and one fungal species. While this collection of species does not represent the full diversity of Ath pathogens, it covers the majority of pathogens for which effector interactors have been reported. We can see that, despite being one of the major pathogen classes, few studies have described fungal effector interactors. This illustrates one of the current gaps in our knowledge of effector interactors in Ath.

### 2.4 Identification of one hundred new “effector hubs”

**FIGURE 3:**
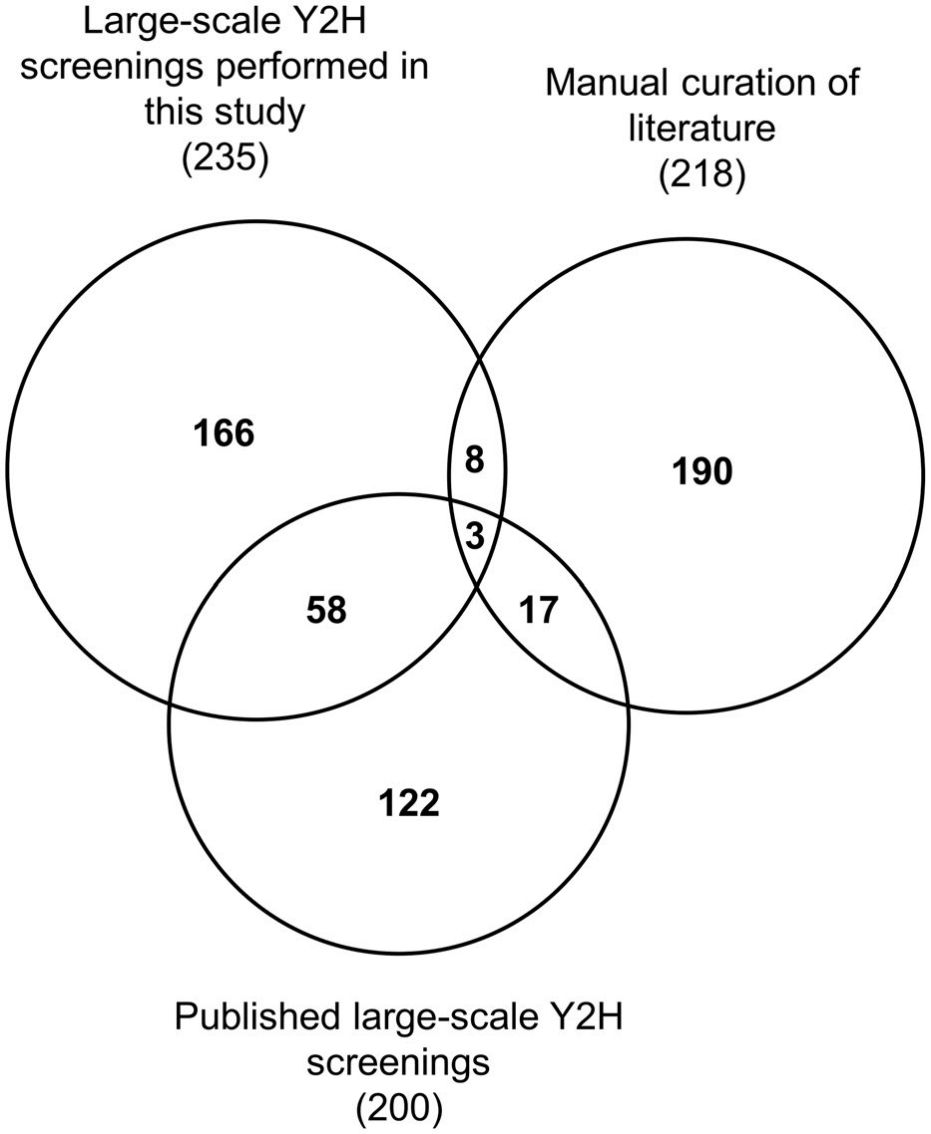
Overlap among effector interactors depending on the origin of the data set. Area‐proportional Venn diagram showing the overlap among effector interactors identified in the large‐scale yeast two‐hybrid (Y2H) screenings performed in this study, in similar large‐scale Y2H already published, and in the manual curation of the literature. The total number of effector interactors coming from each dataset is indicated in parentheses

**FIGURE 4:**
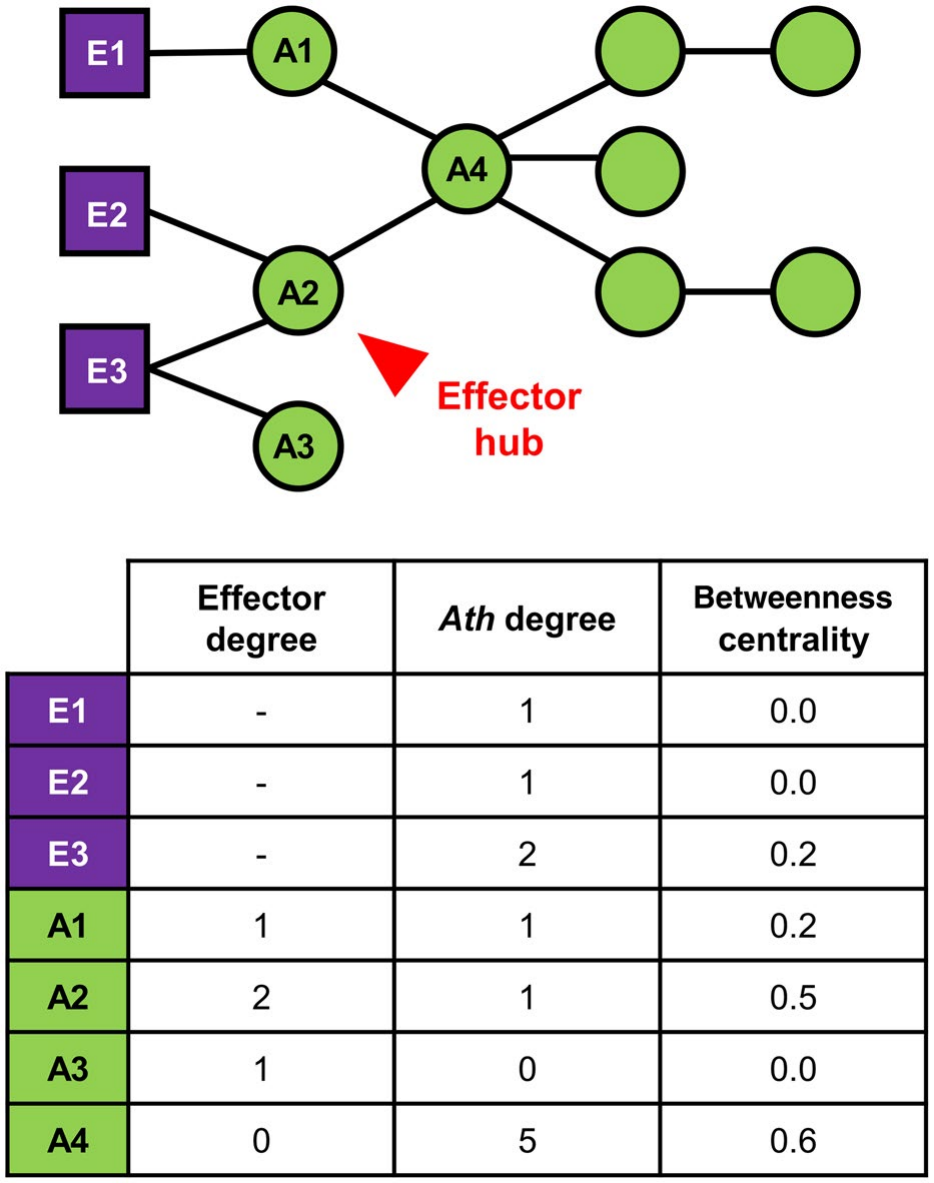
Network topology parameters. Example of a simple interactomic network of three effector proteins (purple squares) and nine *Arabidopsis thaliana* (Ath) proteins (green circles) to illustrate our definition of “effector hub” (i.e., Ath protein interacting with two or more effectors; highlighted in red) and the three network topology parameters analysed in this study. 1, Effector degree: number of effectors that interact with a given Ath protein; 2, Ath degree: number of Ath proteins that interact with a given effector or Ath protein; 3, Betweenness centrality: fraction of all shortest paths connecting two proteins from the network that pass through a given protein

To compare experimental and published data, we combined all the interactions curated from the published data together with data from our large‐scale Y2H screenings. This resulted in a total of 564 different Ath proteins interacting with pathogen effectors. Our screenings on Rps and Xcc effectors identified 235 interactors. Similar published screenings on Psy, Gor, or Hpa effectors had identified 200 interactors (Mukhtar *et al*., 2011; Weßling *et al*., 2014). The literature curation allowed us to identify 218 effector interactors. From the 235 Rps and Xcc effectors interactors found in our screening, 166 were new, which represents 29.4% of the total interactors compiled in this study (Figure 3). This highlights the potential of such systematic and high‐throughput large‐scale screenings in identifying novel effector interactors. The average effector degree (i.e., the number of effectors interacting with a given Ath protein) was 2.3 but it was unevenly distributed among the 564 interactors, with 350 of them interacting with only one effector (62%) and 14 interacting with more than 10 effectors (2.5%) (Figure S4). The contribution of our experimental data was important in the identification of single interactors as we identified 93 out of the 350 (26.6%). More remarkable was the contribution in the identification of “effector hubs,” defined here as Ath proteins interacting with two or more effectors (Figure 4). The definition of “hub” has been debated and it has been traditionally associated with proteins that are highly connected in interactomic networks (Vandereyken *et al*., 2018). Our definition of “effector hub” came from the need to designate the Ath proteins that interact with several effectors and is based exclusively on the number of interacting effector proteins. We identified 100 new effector hubs and increased the degree of 42 previously described effector hubs (Table S3).

To evaluate the potential relevance of the newly identified effector hubs in plant immunity, we conducted a second literature survey to check if the corresponding Ath genes had previously reported functions in plant immunity or in pathogen fitness in planta (Table 1). Sixteen out of the 100 new effector hub genes have already been described for their altered infection or other immunity‐related phenotype when mutated, silenced or overexpressed. Additionally, the orthologs of three other new hubs in other plant species also produced altered infection phenotypes when silenced or overexpressed. A total of 19 out of the 100 newly identified effector hubs have already been shown to be involved in biotic stress responses. Considering that many of the remaining newly defined effector hubs have been poorly characterized (e.g., hypothetical proteins or descriptions based on homology or belonging to a protein family), it is likely that the number of effector hubs involved in immunity was underestimated. This constitutes a valuable source of novel candidates for further functional characterization.

**TABLE 1:** List of 19 new effector hubs involved in plant immunity

Effector hub	Protein name	Effector degree^a^	Description of observed phenotype	Reference
AT1G58100	TCP domain protein 8 (TCP8)	13	Triple *tcp8 tcp14 tcp15* mutant showed enhanced *Pseudomonas syringae* strain DC3000 ∆*avrRps4* growth	Kim *et al*. (2014)
AT1G71230^b^	COP9‐signalosome 5B (CSN5B)	8	Wheat *TaCSN5* mutant showed enhanced disease symptoms caused by *Puccinia triticina*	Zhang *et al*. (2017)
AT3G12920	BOI‐related gene 3 (BRG3)	7	*brg3* mutant showed increased *Botrytis cinerea* lesion size	Luo *et al*. (2010)
AT5G08330^b^	TCP domain protein 21 (TCP21)	7	Rice *OsTCP21* silenced and overexpressing plants showed enhanced and reduced disease symptoms caused by rice rust stunt virus (RRSV), respectively	Zhang *et al*. (2016)
AT5G61010	Exocyst subunit EXO70 family protein E2 (EXO70E2)	6	*exo70e2* mutant showed reduced flg22‐induced callose deposition.	Redditt *et al*. (2019)
AT4G00270	STOREKEEPER‐related 1 (STKR1)	6	*STKR1* overexpressing plants showed reduced *Hyaloperonospora arabidopsidis* spore formation	Nietzsche *et al*. (2018)
AT3G01670	SIEVE ELEMENT OCLUSSION‐related 2 (SEOR2)	4	*Myzus persicae* feeding from *seor2* mutant showed reduced progeny	Anstead *et al*. (2012)
AT5G17490	RGA‐like protein 3 (RGL3)	3	*rgl3* mutant showed reduced *P. syringae* growth and increased SA content upon infection	Li *et al*. (2019)
AT3G54230	Suppressor of *abi3‐5* (SUA)	3	*sua* mutant showed enhanced *P. syringae* growth and reduced chitin‐induced ROS production	Zhang *et al*. (2014)
AT3G11410	Protein phosphatase 2CA (PP2CA)	3	*pp2ca* mutant showed reduced *P. syringae* colonization and stomatal aperture. *PP2CA* overexpressor showed enhanced stomatal aperture	Lim *et al*. (2014)
AT2G17290	Calcium‐dependent protein kinase 6 (CPK6)	3	Double *cpk5‐cpk6* mutant showed enhanced *P. syringae* growth and reduced flg22‐induced ROS production	Boudsocq *et al*. (2010)
AT5G41410^b^	Homeobox protein BEL1 homolog (BELL1)	3	Rice *OsBIHD1* mutant and overexpressing plants showed increased and reduced *Magnaporthe oryzae* lesion area, respectively	Liu *et al*. (2017)
AT4G26750	LYST‐interacting protein 5 (LIP5)	2	*lip5* mutant showed enhanced *P. syringae* growth and disease symptoms and reduced endosomal structure formation upon infection	Wang *et al*. (2014)
AT4G35090	Catalase‐2 (CAT2)	2	*cat2* mutant showed increased ROS accumulation upon infection with incompatible *P. syringae* strain	Simon *et al*. (2010)
AT3G02870	Inositol‐phosphate phosphatase (VTC4)	2	*vtc4* mutant showed reduced *P. syringae* growth	Mukherjee *et al*. (2010)
AT5G53060	Regulator of CBF gene expression 3 (RCF3)	2	*rcf3* mutant showed reduced percentage of diseased plants and higher percentage of plant survival upon *Fusarium oxysporum* infection	Dagdas *et al*. (2016)
AT3G02540	RAD23 family protein C (RAD23C)	2	*rad23BCD* mutant (and not *rad23BD*) did not show *Candidatus Phytoplasma*‐induced flower virescence and phyllody	MacLean *et al*. (2014)
AT5G38470	RAD23 family protein D (RAD23D)	2	*rad23D* mutant did not show flower virescence and phyllody upon transgenic expression of *C. phytoplasma* SAP54 effector	MacLean *et al*. (2014)
AT2G37630	Asymmetric leaves 1 (AS1)	2	*as1* mutant showed reduced lesion size caused by *B. cinerea* and *Alternaria brassicicola* and enhanced *Pseudomonas fluorescens* and *P. syringae* growth	Nurmberg *et al*. (2007)

^a^ Ranked in decreasing order.

^b^ Orthologous gene in other plant species, as defined by EnsemblPlants (Kersey *et al*., 2018), characterized for a role in immunity.

In terms of organism of origin, most of the 564 interactors are bacterial effector interactors, as could be expected considering that 132 out of the 266 total effectors compiled came from bacteria (Figure S4). In the case of effector hubs, it is noteworthy that 133 out of the 214 hubs described in this work interact with effectors from a single kingdom while there are only 64, 16, and one hubs interacting with effectors from two, three or four different kingdoms, respectively (Table S3). Although biased by the structure of the data, this could suggest kingdom specificity of effector targeting.

### 2.5 Construction of an interaction network involving *Arabidopsis* and effector proteins

We constructed an Ath–effector protein interaction network compiling the previously described experimental and literature‐compiled data with Ath–Ath protein interactions from public databases and the literature (Stark *et al*., 2006; Dreze *et al*., 2011; Orchard *et al*., 2014; Smakowska‐Luzan *et al*., 2018). From the total of 49,500 interactions compiled in this study, 48,597 were grouped into a single connected component constituting what we defined as our Ath–effector interactomic network (Table S4). This network was constituted of 47,314 Ath–Ath and 1,283 Ath–effector protein interactions between 8,036 Ath proteins and 245 effector proteins. Effectors came from 23 different species, including bacteria (128 effectors), oomycetes (61 effectors), fungi (46 effectors), and invertebrates (10 effectors). The uneven distribution of effectors among kingdoms highlights the contribution of the large‐scale screenings in the identification of effector interactors as 1,002 out of 1,283 Ath–effector protein interactions came from either our experimental data or previous screenings of the same library (Mukhtar *et al*., 2011; Weßling *et al*., 2014).

### 2.6 Effector interactors tend to occupy key positions in the *Arabidopsis–*effector protein interaction network

**FIGURE 5:**
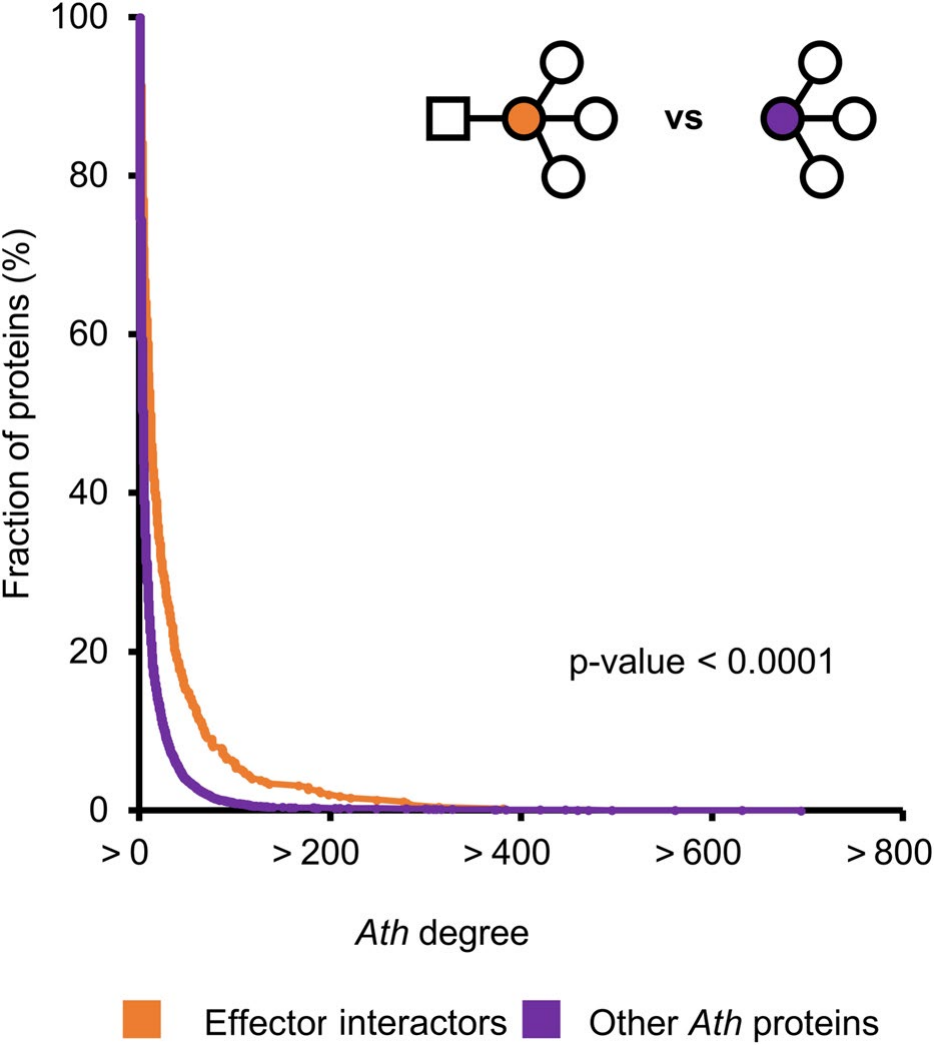
Ath degree of Ath proteins interacting or not with effectors. Cumulative distribution of Ath degree of Ath proteins interacting (orange) or not (purple) with effectors. The significance of the difference was validated by one‐tailed Wilcoxon signed‐rank test. The illustration in the upper right corner represents each compared group. Effectors are represented by squares, Ath proteins by circles and the colour code matches the cumulative distribution graph

To further investigate the potential impact of effectors on the plant interactome, we evaluated the importance of their interactors for the organization of the network. We focused on two main network topology parameters: “degree” and “betweenness centrality” (Figure 4). The “degree” of a protein represents the number of proteins that it interacts with. In this study we differentiated two types of degrees depending on the nature of the interacting proteins: the Ath degree of a given effector or Ath protein (i.e. the number of interacting Ath proteins) and the effector degree for a given Ath protein (i.e. the number of interacting effector proteins). The “betweenness centrality” of a protein is the fraction of all shortest paths connecting two proteins from the network that pass through it. There are two main types of key proteins in a network (Li *et al*., 2017): (a) proteins important for local network organization, typically showing high degree, and (b) proteins important for the global diffusion of the information through the network, characterized by high betweenness centrality. It had been previously reported in more limited networks that effectors tend to interact with host proteins with high degree and high centrality (Memišević *et al*., 2015; Li *et al*., 2017; Ahmed *et al*., 2018). We then analysed whether this was the case in our network comparing effector interactors with the rest of the Ath proteins (Figure 5). The fraction of proteins decreased rapidly as the Ath degree increased. This indicates that most Ath proteins present low Ath degree and only a few of them show high Ath degree values. This tendency was significantly shifted towards higher Ath degree values in effector interactors compared to the rest of Ath proteins. To represent this tendency shift we estimated and compared the area under the curve values of the cumulative distribution of the Ath degree for effector interactors and the rest of Ath proteins (Table 2). Effectively, the area under the curve value of effector interactors was higher than the value of the rest of the Ath proteins. This indicates that effector interactors present generally higher Ath degree than the rest of the Ath proteins. Similarly, we compared the betweenness centrality of these two groups of proteins (Table 2 and Figure S5). Effector interactors also presented significantly higher betweenness centrality values than the rest of the Ath proteins. Altogether, these results indicate that effectors preferentially interact with Ath proteins that are more connected to other Ath proteins and that occupy more central positions in the interactomic network as reported for smaller networks (Li *et al*., 2017; Ahmed *et al*., 2018).

**TABLE 2:** Cumulative *Ath* and effector degrees and betweenness centrality of different groups of effector interactors

	Area under the curve^a^		Figure^b^	*p* value^c^
	**Effector interactors**	**Other *Ath* proteins**		
*Ath* degree	2,737	1,010	5	<.0001
Betweenness centrality	0.23	0.033	S5A	<.0001
	**Multipathogen effector interactors**	**Pathogen‐specific effector interactors**		
*Ath* degree	5,344	1,790	S5B	<.0001
Betweenness centrality	0.657	0.136	S5C	<.0001
	**Effector hubs**	**Single effector interactors**		
*Ath* degree	4,067	1,810	S5D	<.0001
Betweenness centrality	0.407	0.118	S5E	<.0001
	**Bacterial core T3Es**	**Rest of bacterial T3Es**		
*Ath* degree	656	712	S7A	0.4571
Betweenness centrality	0.072	0.074	S7B	0.9198
	**Bacterial core T3E interactors**	**Other bacterial T3Es interactors**		
Effector degree	347	123	S7C	<.0001
*Ath* degree	3,610	2,714	S7D	0.0131
Betweenness centrality	0.369	0.239	S7E	0.0007

^a^ Estimated area under the curve of the cumulative distribution of *Ath* degree, effector degree, and betweenness centrality for each group of proteins as represented in Figures 5, S5, and S7. Estimation based on numerical integration using Simpson's rule.

^b^ Figure illustrating the cumulative distribution graphic from which the areas under the curve compared were calculated.

^c^ One‐tailed Wilcoxon signed‐rank test *p* value of the comparison of the *Ath* degree, effector degree or betweenness centrality values of all proteins from each compared group.

### 2.7 Effector hubs are better connected and more central than single effector interactors in the *Arabidopsis–*effector interaction network

We then wanted to test if the Ath d*e*gree and betweenness centrality values differed among distinct types of effector interactors (Table 2 and Figure S5). First, we compared multipathogen and pathogen‐specific interactors as previously described (Figure S2). Multipathogen effector interactors presented significantly higher Ath degree and betweenness centrality compared to pathogen‐specific effector interactors. We also compared effector hubs with single effector interactors. Similarly, effector hubs also showed higher betweenness centrality and Ath degree than single effector interactors. This last observation implies that an Ath protein that interacts with several effectors tends also to interact with more Ath proteins. To evaluate whether this is biologically relevant or a bias of the “stickiness” of a protein, we compared the Ath and effector degree values of all effector interactors. Our results showed that these two parameters are not correlated (Pearson correlation coefficient = 0.3221; Figure S6). This suggests that effector hubs interact with more Ath proteins than single effector interactors and that this is not due to a higher stickiness of these proteins. Altogether, these results show that the general tendencies of effector interactors (i.e. more connected to other Ath proteins and more central in the *Arabidopsis–*effector interaction network) are stronger among effector hubs compared to single interactors, and among multipathogen effector interactors compared to pathogen‐specific interactors. This reflects the importance of interfering with key position proteins for the modulation of host–pathogen interactions.

### 2.8 Bacterial core T3Es interact with more connected and central Ath proteins

Our work on Rps and Xcc together with previous work on Psy T3Es (Mukhtar *et al*., 2011) provided a large amount of interactomic data on bacterial pathogen species for which other resources have been generated, particularly in terms of abundance and diversity of sequenced genomes and thus curated T3E repertoires (Lindeberg *et al*., 2012; Guy *et al*., 2013; Peeters *et al*., 2013; Roux *et al*., 2015; Dillon *et al*., 2019; Sabbagh *et al*., 2019). The most conserved set of T3Es, or “core effectome,” from each of the three bacterial species has been previously defined (Guy *et al*., 2013; Dillon *et al*., 2019; Sabbagh *et al*., 2019). We then tested whether these subsets of T3Es behaved differently from the rest of bacterial T3Es in terms of interaction with host proteins (Table 2 and Figure S7). Our data showed that core and variable T3Es from the three species do not differ in Ath degree nor betweenness centrality. We then tested if there were any differences between the network properties of the interactors of core T3Es and the other bacterial T3E interactors. Core T3Es interactors showed higher effector degree, Ath degree, and betweenness centrality than the rest of interactors of bacterial T3Es. This suggests that, although core T3Es in general do not have more interactors than the rest of bacterial T3Es, they do interact with more highly connected and central Ath proteins. This might imply that core T3Es have a larger potential to interfere with the host interactome, which could explain the selective pressure to maintain them in the majority of strains.

### 2.9 EffectorK, an online interactive knowledge database to explore the *Arabidopsis–*effector interactomic data

**FIGURE 6:**
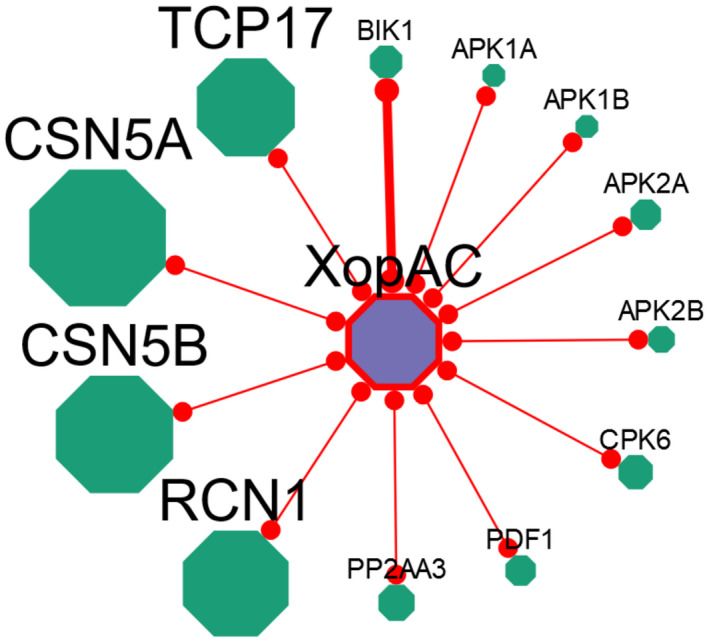
Graphical representation of interactomic data on EffectorK. Graphical representation of interactomic data from *Xcc* effector XopAC (AvrAC). XopAC, in purple, interacts with 36 Ath proteins, in green (only 12 shown for better visualization). The size of a protein node is proportional to its degree (e.g. CSN5B interacts with 50 proteins, BIK1 with six, and APK1A only with XopAC). The thickness of the connecting edges indicates the level of confidence: narrow edges represent physical interaction detected by only one technique, whereas thick edges indicate that the interaction has been detected by at least two independent techniques (e.g. XopAC interaction with BIK1 has been detected by co‐immunoprecipitation and pulldown assays, whereas the interaction with APK1A, only by Y2H)

In order to facilitate the access and exploration of all the data presented in this work, we have generated EffectorK (for “Effector Knowledge”), an interactive web‐based knowledge database freely available at www.effectork.org. The latest version (2 October 2019) contains 49,875 interactions for 8,617 proteins coming from 2,035 publications. Of these, 1,300 are Ath–effector protein interactions. Searches can be done based on a wide range of supported identifiers such as different protein names, NCBI or TAIR accession numbers, PubMed identifiers, and InterPro terms. Additionally, users can also query nucleotide or amino acid sequences directly with BLAST or use accession numbers from other model and crop plants to find homologs within the database. All proteins found by query are then listed in tabular format and hyperlinked to the corresponding interactomic data, external resources, and amino acid sequences. Interactomic data for a given protein can be then explored and downloaded in tabular or graphical format. The graphical representation of the interactomic data depicts proteins interacting with other proteins as nodes interconnected by edges (Figure 6). The size of a node is proportional to the number of interacting proteins, whereas the thickness of an edge represents the confidence of the interaction (i.e. whether the interaction has been detected by one [narrow] or several independent [thick] techniques). This visual interface allows users to expand or re‐centre a local subnetwork based on a given protein, get information and access to external resources linked to either a protein (node) or an interaction (edge), or modify the layout and the position of the elements for optimal visualization. Additionally, EffectorK also allows users to find the shortest paths between two queried proteins in the network.

In order to update, expand, and further improve EffectorK, we encourage users to submit their own interactomic data by filing in and sending a dedicated template available on the site. These data will be verified by the curator team prior to their incorporation in the database. More information about usage, content, and data submission is accessible online, under the tabs “Help” and “Contribute” of the database web server. Please contact us if you have any question or suggestions by email via contact@effectork.org.

## 3 DISCUSSION

In this study we identified systematically Ath interactors of effectors from the vascular bacterial pathogens Rps and Xcc. We combined this information with other Ath interactors identified in similar experimental setups. Additionally, we conducted an extensive literature review to gather published Ath interactors of effectors from a wide variety of pathogens, including other bacterial species and also oomycete, fungal, and invertebrate pathogens. Studying this combined interactomic dataset allowed us to identify new trends of how effectors interfere with the plant proteome and evaluate whether previously described network principles were still supported on a wider scale. We showed that there are no substantial differences in terms of connectivity among the effectomes of five different pathogen species screened systematically (Figure 1). We have reinforced previously described intra‐ and interspecific convergence of effector targeting with effectors from two new species (Mukhtar *et al*., 2011; Weßling *et al*., 2014), and showed at the same time that most effector interactors are pathogen specific (Figure 2 and S2). Our analyses also supported the previously described tendency of effectors to interact with plant proteins better connected and central in the network (Li *et al*., 2017; Ahmed *et al*., 2018), and showed that this tendency is even stronger among effector hubs, multipathogen interactors, and bacterial core T3E interactors (Table 2 and Figure S5).

### 3.1 The balance between interactor specificity and convergence

Our data showed that most effector interactors were pathogen‐specific (Figure S2) but at the same time effectors converge interspecifically onto a small subset of Ath proteins (Figure 2B,C). These a priori contradictory observations pose an interesting question: what is the balance between the specificity and convergence of effector interactors? At this point, it is impossible to assert whether this specificity is merely caused by the limited number of pathogens screened at the effectome‐scale or if it is a reflection of the different and unique ways that each pathogen has evolved to interfere with the host physiology and immunity. This issue can only be addressed by increasing the number of pathogen effectors screened thoroughly and at a large scale. Comparing large datasets of effector interactors of a wider and more diverse set of pathogens would allow evaluating where the balance is between specificity and convergence: (a) If the interactor specificity decreased, it would mean that the effectomes from the different pathogens tend to interact similarly with the host proteome. This was the case when we compared the percentage of species‐specific interactors of effectors from Hpa, Psy, and Gor that passed from being 73.9%, 64.9%, and 46.7% in previous works (Mukhtar *et al*., 2011; Weßling *et al*., 2014), to 51.7%, 58.9%, and 35.6%, respectively, in the present study (Figure S2). Nevertheless, a total of five screened species is probably not powerful enough to sustain this claim. (b) If, in contrast, the interactor specificity increased with the number of screened species, it would mean that the different pathogens have evolved unique ways to modulate the interaction with the host. If this were the case, deeper analyses comparing related pathogens (e.g. species with similar lifestyle or from the same kingdom) could allow identifying trait‐specific interactors (e.g. effector interactors exclusive among vascular pathogen effectors). In any case, to better understand the similarities and particularities on how effectors modulate host processes, it is essential to increase the number of pathogen species screened for effector interactors at the effectome‐scale.

### 3.2 Large‐scale screenings fill the gap in the identification of effector interactors

Including manually curated data from literature has allowed us to broaden significantly the diversity of plant pathogen species compared to similar studies. However, 346 out the 564 described *Arabidopsis* effector interactors have been identified exclusively through large‐scale Y2H screenings against partial libraries of Ath cDNAs. As with any other large‐scale screening, the technical limitations together with the incompleteness of the library might have led to an underestimation of the plant–effector interactome of the five screened species (Brückner *et al*., 2009). The relatively small overlap between the large‐scale Y2H screenings and manually curated literature data sets might be a consequence of this limitation (Figure 3). This small overlap illustrates the current knowledge gap in the characterization of the full plant interactome of pathogen effectors. Extensive work will be required to characterize further effector–host protein interactions in other pathosystems. As one of the simplest yet powerful high‐throughput techniques for protein–protein interaction detection, our work, like others before, highlights the potential of such large‐scale Y2H screenings in the identification of novel effector interactors in an easy, cheap, and systematic manner.

### 3.3 EffectorK, an entry point to explore and make sense of plant–effector interactomics

To conclude, our work also provides valuable resources for the plant–pathogen interaction community. We described 540 new *Ath–Rps* and *Ath–Xcc* effector protein interactions that allowed us to identify 166 new effector interactors (Table S1). We also manually curated several publications to assemble a collection of 287 Ath–effector protein interactions from a wide variety of pathogens (Table S2). All this allowed us to identify 100 novel effector hubs (Table S3). The contribution to plant immunity of these effector hubs has been described for 19 of them, but remains untested for the majority (Table 1). This constitutes a list of promising candidates for further functional characterization. All these data were integrated in EffectorK, a knowledge database where users can have easy access to the Ath–effector protein interactions and explore the resulting interactomic network visually and interactively. While major efforts were made to capture the maximal diversity on the pathogen side, we limited our work to the *Arabidopsis* plant model. Thanks to the built‐in homology search tools available, users can also use their own data as query regardless of the species studied. It is therefore feasible to use EffectorK as a starting point to build on and extend to crop plant–effector protein interactomics. In the long term, these data could be exploited to better understand how pathogens interact with these crops with the prospect of selecting breeding candidates for improved tolerance or resistance against pathogens.

## 4 EXPERIMENTAL PROCEDURES

### 4.1 Cloning of Rps and Xcc T3E genes

All the cloning of the T3E genes from Rps and Xcc was performed by BP gateway BP or TOPO cloning (Thermo Fisher Scientific, Waltham, MA, USA) to generate pENTRY plasmids, which were later transferred into the appropriate Y2H plasmids (Mukhtar *et al*., 2011) using the LR gateway reaction (Thermo Fisher Scientific). Table S5 contains all the PCR primers and final plasmid identities describing the collection of plasmids used in this study. Gene sequence information from Rps strain GMI1000 (GenBank accessions: NC_003295 and NC_003296) (Salanoubat *et al*., 2002) can be obtained from www.ralsto‐T3E.org (Sabbagh *et al*., 2019) and from the published genome of Xcc strain 8,004 (NC_007086) (Qian, 2005).

### 4.2 Y2H screenings

The Y2H screening was performed in semi‐liquid (“8K space” screening) and liquid (“12K space” screening) media as recently reported (Monachello *et al*., 2019), which is an adaptation of a previously developed Y2H‐solid pipeline (Dreze *et al*., 2010). In both protocols the same low copy number yeast expression vectors and the two yeast strains, *Saccharomyces cerevisiae* Y8930 and Y8800, were used. The expression of the *GAL1‐HIS3* reporter gene was tested with 1 mM 3AT (3‐amino‐1,2,4‐triazole, a competitive inhibitor of the HIS3 gene product) unless described otherwise. Prior to Y2H screening, DB‐X strains were tested for auto‐activation of the *GAL1‐HIS3* reporter gene in the absence of AD‐Y plasmid. In case of auto‐activation, DB‐X were physically removed from the collection of baits and screened against the (DB)‐Ath‐cDNA collections using their AD‐X constructs. Briefly, DB‐X baits expressing yeasts were individually grown (30 °C for 72 hr) in 50‐ml polypropylene conical tubes containing 5 ml of fresh selective media (Sc−leucine, Sc−Leu). Pools were created by mixing a maximum of 72 and 50 individual bait yeast strains for the “8K space” and “12K space”, respectively. Subsequently, 120 and 50 µl of these individual pools were plated into 96‐well and 384‐well low‐profile microplates for Ath‐cDNA “8K space” and “12K space” collections, respectively. Glycerol stocks of the (AD)‐Ath‐cDNA “8K space” and “12K space” collections were thawed, replicated by handpicking or using a colony picker Qpix2 XT into 96‐well and 384‐well plates filled with 120 and 50 µl of fresh selective media (Sc−tryptophan, Sc−Trp), respectively, and incubated at 30 °C for 72 hr. Culture plates corresponding to the DB‐baits pools and AD‐collection were replicated into mating plates filled with YEPD media and incubated at 30 °C for 24 hr. In liquid Y2H case (“12K space” screening), mating plates were then replicated into screening plates filled with 50 µl of fresh Sc−Leu−Trp−histidine + 1 mM 3AT media and incubated at 30 °C for 5 days. In order to identify primary positives, the OD_600_ of the 384‐well screening plates was measured using a microplate reader Tecan Infinite M200 PRO (Tecan, Männedorf, Switzerland). In semi‐liquid Y2H case (“8K space” screening), mated yeast were spotted onto Sc−Leu−Trp−histidine + 1 mM 3AT media agar plates, and incubated at 30 °C for 3 days. Protein pairs were identified by depooling of DB‐baits in a similar targeted matricial liquid or semi‐liquid assays in which all the DB‐baits were individually tested against all the previously identified AD‐proteins. Identified pairs were picked and checked by PCR and DNA sequencing.

### 4.3 Database content and manual curation

Binary interactions between Ath proteins with each other and with pathogen effector proteins were compiled on tabular form keeping track of the protein names and accessions, species and ecotypes/strains of origin, techniques used to detect the interactions and the reference. Ath–Ath protein interactions were compiled from the Arabidopsis Interactome (Dreze *et al*., 2011; Smakowska‐Luzan *et al*., 2018) and the public databases BioGrid (www.thebiogrid.org [Stark *et al*., 2006], downloaded in September 2019) and IntAct (www.ebi.ac.uk/intact [Orchard *et al*., 2014], downloaded in September 2019). We only kept the direct interactions with the evidence codes “co‐crystal structure,” “FRET” (fluorescence resonance energy transfer), “PCA” (protein‐fragment complementation assay), “reconstituted complex” or “two‐hybrid” on BioGrid and “physical association” on IntAct. Ath*–*effector protein interactions were gathered from our experimental Y2H data together with the similarly produced data on Hpa, Psy, and Gor effectors (Mukhtar *et al*., 2011; Weßling *et al*., 2014). In addition, an extensive keyword search on effector–*Arabidopsis* literature was done to retrieve interactions from 80 published articles. A confidence level was assigned to each interaction depending on the number of independent techniques used in a publication for validation: “1” if the interaction was detected by only one technique and “2” if the interaction was validated by at least a second technique. Some interactions lacked important information but, in order to maximize the extent of our network, several assumptions were taken instead of discarding useful data. First, gene models for Ath proteins were rarely mentioned on publications so we assumed the first gene model available on the latest version of the *Arabidopsis* genome (Araport11 (Cheng *et al*., 2017)). Second, when the ecotype/strain of the organism was not explicitly stated, a generic “NA” (not available) was assigned.

### 4.4 In silico analysis

#### 4.4.1 Computational simulations of random targeting of Ath proteins by single pathogen effectors (intraspecific convergence)

Significance of the intraspecific convergence was tested, comparing our experimental data with random simulations as previously published (Weßling *et al*., 2014). Briefly, for each effector of Xcc and Rps we assigned randomly the same number of Ath interactors as experimentally observed from the degree‐preserved list of 8K proteins. The distribution obtained from 10,000 simulations was plotted and compared to the experimentally obtained data. The *p* value of the experimental data were calculated as follows: number of simulations where the number of interactors is lower than or equal to experimentally observed is divided by the number of simulations. When the number of simulations with fewer interactors than observed was zero, the *p* value was set to <.0001.

(1) $$p\;\text{value}\;=\;\frac{\text{number of simulations where the number of interactors}\;\leq \;\text{experimentally observed number of interactors}}{\text{number of simulations}}$$

#### 4.4.2 Computational simulations of random targeting of Ath proteins by several pathogen effectors (interspecific convergence)

The significance of the interspecific convergence was tested by comparing our experimental data and previously published data with random simulations as published (Mukhtar *et al*., 2011; Weßling *et al*., 2014). Briefly, for each effector of all compared pathogens we assigned the same number of Ath interactors as experimentally observed/published from the list of 8K proteins. The distribution obtained from 10,000 simulations was plotted and compared to experimentally and published data. The *p* values of the experimental data were calculated as follows: number of simulations where the number of common interactors between species was higher or equal than the experimentally observed is divided by the number of simulations. When the number of simulations with more common interactors than observed was zero, the *p* value was set to <.0001.

(2) $$p\;\text{value}\;=\;\frac{\text{number of simulations where the number of common interactors}\;\geq \;\text{experimentally observed number of common interactors}}{\text{number of simulations}}$$

#### 4.4.3 Overlap of effector interactors

The overlap of effector interactors from the different datasets was calculated without limiting the screening space. For representation of the data, Venn diagrams were generated using the Venn Diagrams tool from VIB‐UGent Center for Plant Systems Biology (www.bioinformatics.psb.ugent.be/webtools/Venn/). The overlap of effector interactors from the different datasets were calculated not limiting to any limited space. For an area‐proportional representation of the data, a Venn diagram was generated using BioVenn (Hulsen *et al*., 2008).

#### 4.4.4 Network topology analyses

The topology parameters of the Ath–effector interactomic network were calculated on Cytoscape 3.7.2 (Shannon, 2003). Our analyses focused on two key node parameters: degree and betweenness centrality. The degree of a protein is a measure of its connectivity and denotes the number of proteins interacting with it. Throughout this work, we have differentiated two kinds of degrees: (a) effector degree (i.e. number of interacting effector proteins) and (b) Ath degree (i.e. number of interacting Ath proteins). The betweenness centrality measures the proportion of shortest pathways between two proteins that passes through a given node. These parameters were compared against different subsets of data and statistical tests were performed in R language (R Core Team, 2019). The cumulative distributions of these parameters among different subset of data were plotted and the area under the curve was estimated using Simpson's rule with the “Bolstad2” package (Bolstad, 2009).

### 4.5 Database construction

The databases were built using the software architecture recently described (Carrère *et al*., 2019). The files submitted by the curator team were automatically checked for typographic mistakes using ad hoc Perl scripts and loaded into a Neo4J database and indexed in an ElasticSearch search engine. Each release was rebuilt from scratch. Data were made accessible through a web interface (see Results and Discussion) built on Cytoscape.js library (Franz *et al*., 2016). The raw data used for the database setup are available in the “Data” section of www.effectork.org and the source code is available at https://framagit.org/LIPM‐BIOINFO/KGBB.

## CONFLICT OF INTEREST

None of the authors has a conflict of interest to declare.

## Supporting information


**FIGURE S1** Ath degree of T3E proteins from Rps strain GMI1000 and Xcc strain 8004

**FIGURE S2** Overlap of Ath interactors of effector proteins from Hpa, Psy, Gor, Rps, and Xcc

**FIGURE S3** Interspecific convergence *of Psy* and* Xcc* effector proteins

**FIGURE S4** Effector degree distribution for Ath effector interactors

**FIGURE S5** Ath degree and betweenness centrality of different groups of Ath effector interactors

**FIGURE S6** Ath and effector degree of effector interactors

**FIGURE S7** Degrees and betweenness centrality of bacterial core and noncore T3Es and their interactors

**TABLE S1** List of Rps and Xcc effector–Ath protein interactions detected experimentally in this study and composition of the Ath‐cDNA screening libraries

**TABLE S2** List of manually curated Ath–effector protein interactions from the literature

**TABLE S3** List of effector hubs and single effector interactors identified

**TABLE S4** List of protein interactions constituting the Ath*–*effector interactomic network

**TABLE S5** List of pENTRY for T3E genes from Rps and Xcc

## Data Availability

The data that support the findings of this study are openly available in EffectorK at www.effectork.org.

## References

[mpp12965-bib-0001] Ahmed, H. , Howton, T.C. , Sun, Y. , Weinberger, N. , Belkhadir, Y. and Mukhtar, M.S. (2018) Network biology discovers pathogen contact points in host protein–protein interactomes. Nature Communications, 9, 2312. 10.1038/s41467-018-04632-8 PMC5998135 29899369

[mpp12965-bib-1004] Anstead, J.A. , Froelich, D.R. , Knoblauch, M. and Thompson, G.A. (2012) Arabidopsis P‐protein filament formation requires both AtSEOR1 and AtSEOR2. Plant & Cell Physiology, 53(6), 1033–1042. 10.1093/pcp/pcs046. 22470058

[mpp12965-bib-0002] Arlat, M. , Gough, C.L. , Barber, C.E. , Boucher, C. and Daniels, M.J. (1991) *Xanthomonas campestris* contains a cluster of *hrp* genes related to the larger *hrp* cluster of *Pseudomonas solanacearum* . Molecular Plant‐Microbe Interactions, 4(6), 593–601. 10.1094/mpmi-4-593 1666525

[mpp12965-bib-0003] Arlat, M. , Gough, C.L. , Zischek, C. , Barberis, P.A. , Trigalet, A. and Boucher, C.A. (1992) Transcriptional organization and expression of the large *hrp* gene cluster of *Pseudomonas solanacearum* . Molecular Plant‐Microbe Interactions, 5(2), 187 10.1094/MPMI-5-187 1617200

[mpp12965-bib-0004] Bolstad, W.M. (2009) Understanding Computational Bayesian Statistics. Hoboken, NJ, USA: John Wiley & Sons, Inc 10.1002/9780470567371

[mpp12965-bib-1008] Boudsocq, M. , Willmann, M.R. , McCormack, M. , Lee, H. , Shan, L. , He, P. *et al* (2010) Differential innate immune signalling via Ca(2+) sensor protein kinases. Nature, 464(7287), 418–422. 10.1038/nature08794. 20164835 PMC2841715

[mpp12965-bib-0005] Brückner, A. , Polge, C. , Lentze, N. , Auerbach, D. and Schlattner, U. (2009) Yeast two‐hybrid, a powerful tool for systems biology. International Journal of Molecular Sciences, 10(6), 2763–2788. 10.3390/ijms10062763 19582228 PMC2705515

[mpp12965-bib-0006] Buell, C.R. (2002) Interactions between Xanthomonas species and *Arabidopsis thaliana* . The Arabidopsis Book, 1, e0031 10.1199/tab.0031 22303203 PMC3243383

[mpp12965-bib-0007] Büttner, D. (2016) Behind the lines‐actions of bacterial type III effector proteins in plant cells. FEMS Microbiology Reviews, 40(6), 894–937. 10.1093/femsre/fuw026 28201715 PMC5091034

[mpp12965-bib-0008] Carrère, S. , Verdenaud, M. , Gough, C. , Gouzy, J. and Gamas, P. (2019) LeGOO: an expertized knowledge database for the model legume *Medicago truncatula* . Plant and Cell Physiology, 10.1093/pcp/pcz177 31605615

[mpp12965-bib-0009] Cheng, C.Y. , Krishnakumar, V. , Chan, A.P. , Thibaud‐Nissen, F. , Schobel, S. and Town, C.D. (2017) Araport11: a complete reannotation of the *Arabidopsis thaliana* reference genome. The Plant Journal, 89(4), 789–804. 10.1111/tpj.13415 27862469

[mpp12965-bib-0010] Coll, N.S. and Valls, M. (2013) Current knowledge on the *Ralstonia solanacearum* type III secretion system. Microbial biotechnology, 6(6), 614–620. 10.1111/1751-7915.12056 23617636 PMC3815929

[mpp12965-bib-0011] Cui, H. , Tsuda, K. and Parker, J.E. (2015) Effector‐triggered immunity: from pathogen perception to robust defense. Annual Review of Plant Biology, 66(1), 487–511. 10.1146/annurev-arplant-050213-040012 25494461

[mpp12965-bib-1013] Dagdas, Y.F. , Belhaj, K. , Maqbool, A. , Chaparro‐Garcia, A. , Pandey, P. , Petre, B. *et al* (2016) An effector of the Irish potato famine pathogen antagonizes a host autophagy cargo receptor. eLife, 5, e10856 10.7554/eLife.10856. 26765567 PMC4775223

[mpp12965-bib-0012] Deslandes, L. , Pileur, F. , Liaubet, L. , Camut, S. , Can, C. , Williams, K. *et al* (1998) Genetic characterization of RRS1, a recessive locus in *Arabidopsis thaliana* that confers resistance to the bacterial soilborne pathogen *Ralstonia solanacearum* . Molecular Plant‐Microbe Interactions MPMI, 11(7), 659–667. 10.1094/MPMI.1998.11.7.659 9650298

[mpp12965-bib-0013] Dillon, M.M. , Almeida, R.N.D. , Laflamme, B. , Martel, A. , Weir, B.S. , Desveaux, D. *et al*(2019) Molecular evolution of *Pseudomonas syringae* type III secreted effector proteins. Frontiers in Plant Science, 10, 10.3389/fpls.2019.00418 PMC6460904 31024592

[mpp12965-bib-0014] Dreze, M. , Monachello, D. , Lurin, C. , Cusick, M.E. , Hill, D.E. , Vidal, M. *et al* (2010) High‐quality binary interactome mapping In Guthrie C., Fink G. and Weissman J. (Eds.) Methods in Enzymology (Vol. 470, pp. 281–315). San Diego, CA: Academic Press 10.1016/S0076-6879(10)70012-4 20946815

[mpp12965-bib-0015] Dreze, M. , Carvunis, A.‐R. , Charloteaux, B. , Galli, M. , Pevzner, S.J. , Tasan, M. *et al* (2011) Evidence for network evolution in an Arabidopsis interactome map. Science, 333(6042), pp. 601–607. 10.1126/science.1203877 21798944 PMC3170756

[mpp12965-bib-0016] Franz, M. , Lopes, C.T. , Huck, G. , Dong, Y. , Sumer, O. and Bader, G.D. (2016) Cytoscape.js: a graph theory library for visualisation and analysis. Bioinformatics, 32(2), 309–311. 10.1093/bioinformatics/btv557 26415722 PMC4708103

[mpp12965-bib-0017] Giron, D. , Huguet, E. , Stone, G.N. and Body, M. (2016) Insect‐induced effects on plants and possible effectors used by galling and leaf‐mining insects to manipulate their host‐plant. Journal of Insect Physiology, 84, 70–89. 10.1016/j.jinsphys.2015.12.009 26723843

[mpp12965-bib-0018] Guy, E. , Genissel, A. , Hajri, A. , Chabannes, M. , David, P. , Carrere, S. *et al* (2013) Natural genetic variation of *Xanthomonas campestris* pv. *campestris* pathogenicity on Arabidopsis revealed by association and reverse genetics. mBio, 4(3). 10.1128/mBio.00538-12 PMC3685212 23736288

[mpp12965-bib-0019] Hulsen, T. , de Vlieg, J. and Alkema, W. (2008) BioVenn—a web application for the comparison and visualization of biological lists using area‐proportional Venn diagrams. BMC genomics, 9, 488 10.1186/1471-2164-9-488 18925949 PMC2584113

[mpp12965-bib-0020] Jones, J.D.G. and Dangl, J.L. (2006) The plant immune system. Nature, 444(7117), 323–329. 10.1038/nature05286 17108957

[mpp12965-bib-1016] Kersey, P.J. , Allen, J.E. , Allot, A. , Barba, M. , Boddu, S. , Bolt, B.J. *et al* (2018) Ensembl genomes 2018: an integrated omics infrastructure for non‐vertebrate species. Nucleic Acids Research, 46, D802–D808. 10.1093/nar/gkx1011. 29092050 PMC5753204

[mpp12965-bib-0046] Kim, S. H. , Son, G. H. , Bhattacharjee, S. , Kim, H. J. , Nam, J. C. , Nguyen, P. D. T. , *et al.* (2014). The Arabidopsis immune adaptor SRFR1 interacts with TCP transcription factors that redundantly contribute to effector‐triggered immunity. The Plant Journal, 78, 978–989. 10.1111/tpj.12527 24689742

[mpp12965-bib-1005] Li, Y. , Yuhua, Y. , Yilong, H. , Hailun, L. , Ming, H. , Ziyin, Y. *et al* (2019) DELLA and EDS1 form a feedback regulatory module to fine‐tune plant growth‐defense tradeoff in Arabidopsis. Molecular Plant, 12(11), 1485–1498. 10.1016/j.molp.2019.07.006. 31382023

[mpp12965-bib-0021] Li, H. , Zhou, Y. and Zhang, Z. (2017) Network analysis reveals a common host–pathogen interaction pattern in Arabidopsis immune responses. Frontiers Plant Science, 8, 893 10.3389/fpls.2017.00893 PMC5446985 28611808

[mpp12965-bib-1007] Lim, C.W. , Luan, S. and Lee, S.C. (2014) A prominent role for RCAR3‐mediated ABA signaling in response to Pseudomonas syringae pv. tomato DC3000 infection in Arabidopsis. Plant & Cell Physiology, 55(10), 1691–1703. 10.1093/pcp/pcu100. 25063782

[mpp12965-bib-1009] Liu, H. , Dong, S. , Gu, F. , Liu, W. , Yang, G. , Huang, M. *et al* (2017) NBS‐LRR protein Pik‐H4 interacts with OsBIHD1 to balance rice blast resistance and growth by coordinating ethylene‐brassinosteroid pathway. Frontiers in Plant Science, 8, 127 10.3389/fpls.2017.00127. 28220140 PMC5292422

[mpp12965-bib-0022] Lindeberg, M. , Cunnac, S. and Collmer, A. (2012) *Pseudomonas syringae* type III effector repertoires: last words in endless arguments. Trends in Microbiology, 20(4), 199–208. 10.1016/j.tim.2012.01.003 22341410

[mpp12965-bib-0044] Luo, H. , Laluk, K. , Lai, Z. , Veronese, P. , Song, F. and Mengiste, T. (2010) The Arabidopsis botrytis susceptible1 interactor defines a subclass of RING E3 ligases that regulate pathogen and stress responses. Plant Physiology, 154(4), 1766–1782. 10.1104/pp.110.163915. 20921156 PMC2996010

[mpp12965-bib-0023] Ma, W. , Wang, Y. and McDowell, J. (2018) Focus on effector‐triggered susceptibility. Molecular Plant‐Microbe Interactions, 31(1), 5–5. 10.1094/MPMI-11-17-0275-LE 29161216

[mpp12965-bib-0024] Mansfield, J. , Genin, S. , Magori, S. , Citovsky, V. , Sriariyanum, M. , Ronald, P. *et al* (2012) Top 10 plant pathogenic bacteria in molecular plant pathology. Molecular Plant Pathology, 13(6), 614–629. 10.1111/j.1364-3703.2012.00804.x 22672649 PMC6638704

[mpp12965-bib-1014] MacLean, A.M. , Orlovskis, Z. , Kowitwanich, K. , Zdziarska, A.M. , Angenent, G.C. , Immink, R.G. *et al* (2014) Phytoplasma effector SAP54 hijacks plant reproduction by degrading MADS‐box proteins and promotes insect colonization in a RAD23‐dependent manner. PLoS Biology, 12(4), e1001835 10.1371/journal.pbio.1001835. 24714165 PMC3979655

[mpp12965-bib-0025] Memišević, V. , Zavaljevski, N. , Rajagopala, S.V. , Kwon, K. , Pieper, R. , DeShazer, D. *et al* (2015) Mining host‐pathogen protein interactions to characterize *Burkholderia mallei* infectivity mechanisms. PLoS Computational Biology, 11(3), e1004088 10.1371/journal.pcbi.1004088 25738731 PMC4349708

[mpp12965-bib-0026] Monachello, D. , Guillaumot, D. and Lurin, C. (2019) A pipeline for systematic yeast 2‐hybrid matricial screening in liquid culture. 10.21203/rs.2.9948/v1

[mpp12965-bib-1012] Mukherjee, M. , Larrimore, K.E. , Ahmed, N.J. , Bedick, T.S. , Barghouthi, N.T. , Traw, M.B. *et al* (2010) Ascorbic acid deficiency in arabidopsis induces constitutive priming that is dependent on hydrogen peroxide, salicylic acid, and the NPR1 gene. Molecular Plant‐Microbe Interactions: MPMI, 23(3), 340–351. 10.1094/MPMI-23-3-0340. 20121455

[mpp12965-bib-0027] Mukhtar, M.S. , Carvunis, A.‐R. , Dreze, M. , Epple, P. , Steinbrenner, J. , Moore, J. *et al* (2011) Independently evolved virulence effectors converge onto hubs in a plant immune system network. Science, 333(6042), pp. 596–601. 10.1126/science.1203659 21798943 PMC3170753

[mpp12965-bib-1003] Nietzsche, M. , Guerra, T. , Alseekh, S. , Wiermer, M. , Sonnewald, S. , Fernie, A.R. *et al* (2018) STOREKEEPER RELATED1/G‐element binding protein (STKR1) interacts with protein kinase SnRK1. Plant Physiology, 176(2), 1773–1792. 10.1104/pp.17.01461. 29192025 PMC5813543

[mpp12965-bib-1015] Nurmberg, P.L. , Knox, K.A. , Yun, B.‐W. , Morris, P.C. , Shafiei, R. , Hudson, A. *et al* (2007) The developmental selector AS1 is an evolutionarily conserved regulator of the plant immune response. Proceedings of the National Academy of Sciences of the United States of America, 104, 18795–18800. 10.1073/pnas.0705586104. 18003921 PMC2141856

[mpp12965-bib-0028] Orchard, S. , Ammari, M. , Aranda, B. , Breuza, L. , Briganti, L. , Broackes‐Carter, F. *et al* (2014) The MIntAct project–IntAct as a common curation platform for 11 molecular interaction databases. Nucleic Acids Research, 42(Database issue), pp. D358–D363. 10.1093/nar/gkt1115 24234451 PMC3965093

[mpp12965-bib-0029] Peeters, N. , Carrère, S. , Anisimova, M. , Plener, L. , Cazalé, A.‐C. and Genin, S. (2013) Repertoire, unified nomenclature and evolution of the Type III effector gene set in the Ralstonia solanacearum species complex. BMC Genomics, 14, 859 10.1186/1471-2164-14-859 24314259 PMC3878972

[mpp12965-bib-0030] Qian, W. (2005) Comparative and functional genomic analyses of the pathogenicity of phytopathogen *Xanthomonas campestris* pv. *campestris* . Genome Research, 15(6), 757–767. 10.1101/gr.3378705 15899963 PMC1142466

[mpp12965-bib-0031] R Core Team (2019) R: A Language and Environment for Statistical Computing Authors R Core Team. Vienna, Austria Available at https://www.r‐project.org/.

[mpp12965-bib-1002] Redditt, T.J. , Chung, E.H. , Zand Karimi, H. , Rodibaugh, N. , Zhang, Y. , Trinidad, J.C. *et al* (2019). AvrRpm1 functions as an ADP‐ribosyl transferase to modify NOI‐domain containing proteins, including Arabidopsis and soybean RPM1‐interacting protein 4. The Plant cell, tpc.00020.2019. Advance online publication. 10.1105/tpc.19.00020.31548257

[mpp12965-bib-0032] Roux, B. , Bolot, S. , Guy, E. , Denancé, N. , Lautier, M. , Jardinaud, M.‐F. *et al* (2015) Genomics and transcriptomics of *Xanthomonas campestris* species challenge the concept of core type III effectome. BMC Genomics, 16(1), 975 10.1186/s12864-015-2190-0 26581393 PMC4652430

[mpp12965-bib-0033] Sabbagh, C.R.R. , Carrere, S. , Lonjon, F. , Vailleau, F. , Macho, A.P. , Genin, S. *et al* (2019) Pangenomic type III effector database of the plant pathogenic *Ralstonia* spp. PeerJ, 7, e7346 10.7717/peerj.7346 31579561 PMC6762002

[mpp12965-bib-0034] Salanoubat, M. , Genin, S. , Artiguenave, F. , Gouzy, J. , Mangenot, S. , Arlat, M. *et al* (2002) Genome sequence of the plant pathogen *Ralstonia solanacearum* . Nature, 415(6871), 497–502. 10.1038/415497a 11823852

[mpp12965-bib-0035] Shannon, P. , Markiel, A. , Ozier, O. , Baliga, N.S. , Wang, J.T. , Ramage, D. , *et al.* (2003) Cytoscape: a software environment for integrated models of biomolecular interaction networks. Genome Research, 13(11), 2498–2504. 10.1101/gr.1239303 14597658 PMC403769

[mpp12965-bib-0036] Sharpee, W.C. and Dean, R.A. (2016) Form and function of fungal and oomycete effectors. Fungal Biology Reviews, 30(2), 62–73. 10.1016/j.fbr.2016.04.001

[mpp12965-bib-1011] Simon, C. , Langlois‐Meurinne, M. , Bellvert, F. , Garmier, M. , Didierlaurent, L. , Massoud, K. *et al* (2010) The differential spatial distribution of secondary metabolites in Arabidopsis leaves reacting hypersensitively to Pseudomonas syringae pv. tomato is dependent on the oxidative burst. Journal of Experimental Botany, 61(12), 3355–3370. 10.1093/jxb/erq157. 20530195

[mpp12965-bib-0037] Smakowska‐Luzan, E. , Mott, G.A. , Parys, K. , Stegmann, M. , Howton, T.C. , Layeghifard, M. *et al* (2018) An extracellular network of Arabidopsis leucine‐rich repeat receptor kinases. Nature, 553(7688), 342–346. 10.1038/nature25184 29320478 PMC6485605

[mpp12965-bib-0038] Stark, C. (2006) BioGRID: a general repository for interaction datasets. Nucleic Acids Research, 34(Database issue), pp. D535–D539. 10.1093/nar/gkj109 16381927 PMC1347471

[mpp12965-bib-0039] Vandereyken, K. , Van Leene, J. , De Coninck, B. and Cammue, B.P.A. (2018) Hub protein controversy: taking a closer look at plant stress response hubs. Frontiers isn Plant Science, 9, 694 10.3389/fpls.2018.00694 PMC5996676 29922309

[mpp12965-bib-0040] Vieira, P. and Gleason, C. (2019) Plant‐parasitic nematode effectors—Insights into their diversity and new tools for their identification. Current Opinion in Plant Biology, 50, 37–43. 10.1016/j.pbi.2019.02.007 30921686

[mpp12965-bib-1010] Wang, F. , Shang, Y. , Fan, B. , Yu, J.Q. and Chen, Z. (2014) Arabidopsis LIP5, a positive regulator of multivesicular body biogenesis, is a critical target of pathogen‐responsive MAPK cascade in plant basal defense. PLoS Pathogens, 10(7), e1004243 10.1371/journal.ppat.1004243. 25010425 PMC4092137

[mpp12965-bib-0041] Weßling, R. , Epple, P. , Altmann, S. , He, Y. , Yang, L. , Henz, S.R. *et al* (2014) Convergent targeting of a common host protein‐network by pathogen effectors from three kingdoms of life. Cell Host & Microbe, 16(3), 364–375. 10.1016/j.chom.2014.08.004 25211078 PMC4191710

[mpp12965-bib-0042] White, F.F. , Potnis, N. , Jones, J.B. and Koebnik, R. (2009) The type III effectors of Xanthomonas. Molecular Plant Pathology, 10(6), 749–766. 10.1111/j.1364-3703.2009.00590.x 19849782 PMC6640274

[mpp12965-bib-1000] Zhang, C. , Ding, Z. , Wu, K. , Yang, L. , Li, Y. , Yang, Z. *et al* (2016) Suppression of jasmonic acid‐mediated defense by viral‐inducible MicroRNA319 facilitates virus infection in rice. Molecular plant, 9(9), 1302–1314. 10.1016/j.molp.2016.06.014. 27381440

[mpp12965-bib-1006] Zhang, Z. , Liu, Y. , Ding, P. , Li, Y. , Kong, Q. and Zhang, Y. (2014) Splicing of receptor‐like kinase‐encoding SNC4 and CERK1 is regulated by two conserved splicing factors that are required for plant immunity. Molecular Plant, 7(12), 1766–1775. 10.1093/mp/ssu103. 25267732 PMC4261838

[mpp12965-bib-0045] Zhang, H. , Wang, X. , Giroux, M. J. and Huang, L. (2017) A wheatCOP9 subunit 5‐like gene is negatively involved in host response to leaf rust. Molecular Plant Pathology, 18, 125–133. 10.1111/mpp.12467 27581057 PMC6638245

[mpp12965-bib-0043] Zipfel, C. (2014) Plant pattern‐recognition receptors. Trends in Immunology, 35(7), 345–351. 10.1016/j.it.2014.05.004 24946686

