# Supplementary material for: EffectorK, a comprehensive resource to mine for Ralstonia, Xanthomonas, and other published effector interactors in the Arabidopsis proteome
Source: Mol Plant Pathol. 2020 Aug 15;21(10):1257–70. doi: 10.1111/mpp.12965 (PMC7488465; doi:10.1111/mpp.12965)
Supplement: Supplementary file 1 — FIGURE S1 Ath degree of T3E proteins from Rps strain GMI1000 and Xcc strain 8004 [file MPP-21-1257-s001.docx]

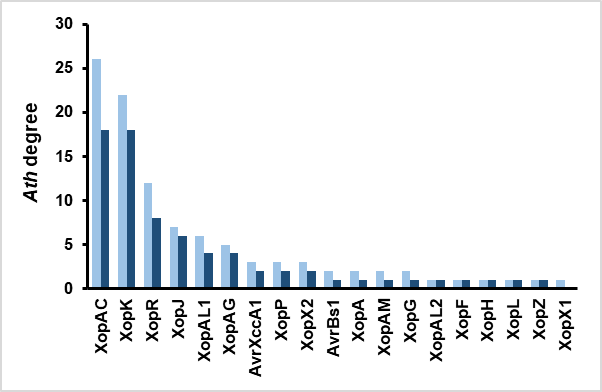

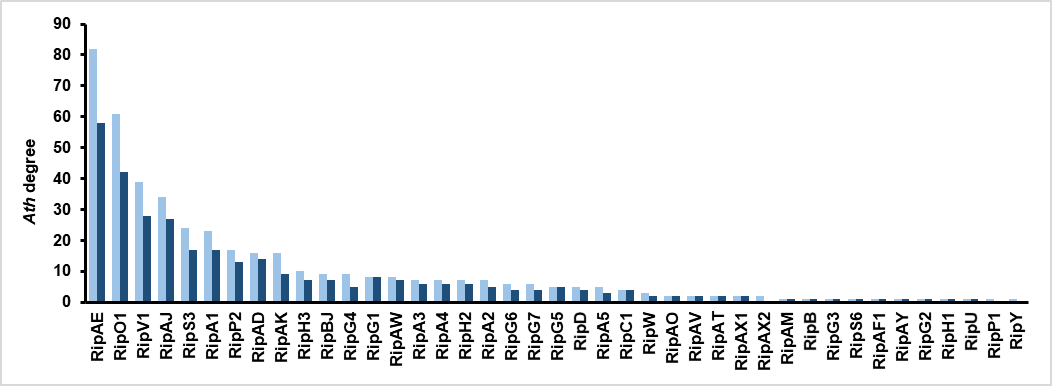


***Rps* strain GMI1000**

12K space

8K space

***Xcc* strain 8004**

12K space

8K space

**Fig S1. *Ath* degree of T3E proteins from *Rps* strain GMI1000 and *Xcc* strain 8004.**

*Ath* degree (i.e., number of *Ath* interactors per effector) in the in the 12,000 (12K space, light blue) and 8,000 *Ath* cDNA collections (8K space, dark blue) of T3E proteins from *Rps* strain GMI1000 (A) and *Xcc* strain 8004 (B). From *Rps* strain GMI1000: in the first screening RipA3, RipAA, RipAB, RipAC, RipAG, RipAL, RipAM, RipAN, RipAO, RipAP, RipAQ, RipAR, RipAZ1, RipB, RipBA, RipG3, RipG4, RipG6, RipG7, RipH2, RipH3, RipI, RipK, RipM, RipN, RipO1, RipP1, RipQ, RipR, RipS2, RipS6, RipT, RipTPS, RipX and RipZ were screened but no interactors were found. In the second screening RipAB, RipAC, RipAO, RipAX1, RipAY, RipBM, RipC1, RipE1, RipH1, RipN, RipR, RipS4, RipU, RipX and RipZ were screened but no interactors were found, and RipAN and RipM could not be screened because of recalcitrant problems with yeast transformation. For *Xcc* strain 8004: AvrXccA2, HpaA, HrpW, XopAN, XopN and XopQ were screened but no interactors were found, and AvrBs2, XopAH, XopAL2, XopD and XopE2 could not be screened because they showed autoactivation in yeast.

**A**

**B**
