# Supplementary material for: EffectorK, a comprehensive resource to mine for Ralstonia, Xanthomonas, and other published effector interactors in the Arabidopsis proteome
Source: Mol Plant Pathol. 2020 Aug 15;21(10):1257–70. doi: 10.1111/mpp.12965 (PMC7488465; doi:10.1111/mpp.12965)
Supplement: Supplementary file 2 — FIGURE S2 Overlap of Ath interactors of effector proteins from Hpa, Psy, Gor, Rps, and Xcc [file MPP-21-1257-s002.docx]

**Fig S2. Overlap of *Ath* interactors of effector proteins from *Hpa*, *Psy*, *Gor*, *Rps* and *Xcc*.**

Venn diagrams showing the overlap among *Ath* interactors found in the 8,000-*Ath*-cDNA collection (8K space) of effector proteins from *Hpa*, *Psy*, *Gor*, *Rps* and *Xcc* at the kingdom (A) and species level (B). The total number of effector interactors for each kingdom/species is indicated in brackets.

**B**

**A**


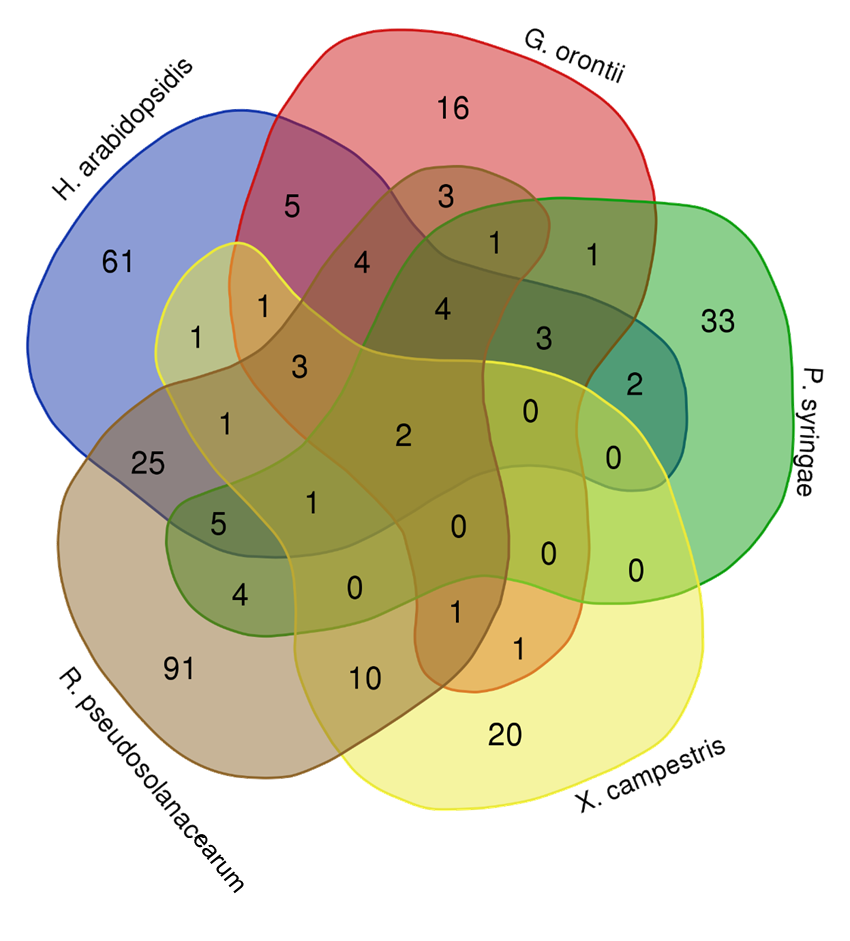


*Hpa* (118)

*Gor* (45)

*Psy* (56)

*Xcc* (41)

*Rps* (155)


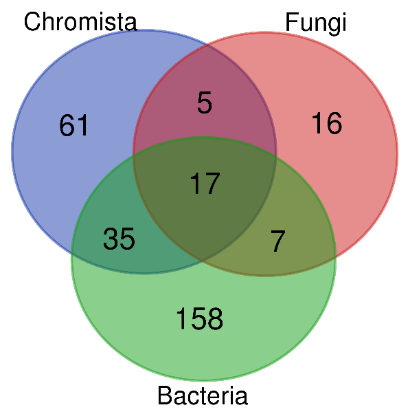


(118)

(45)

(217)
