# Supplementary material for: EffectorK, a comprehensive resource to mine for Ralstonia, Xanthomonas, and other published effector interactors in the Arabidopsis proteome
Source: Mol Plant Pathol. 2020 Aug 15;21(10):1257–70. doi: 10.1111/mpp.12965 (PMC7488465; doi:10.1111/mpp.12965)
Supplement: Supplementary file 3 — FIGURE S3 Interspecific convergence of Psy and Xcc effector proteins [file MPP-21-1257-s003.docx]

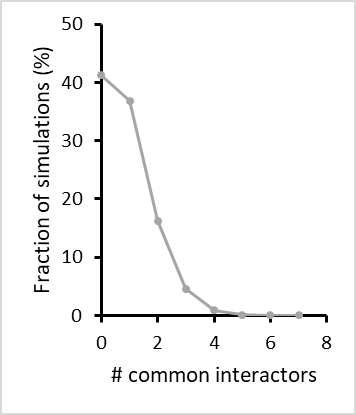


Obs.

(p = 0.0564)

**Fig S3. Interspecific convergence of *Psy* and *Xcc* effector proteins.**

Number of *Ath* interactors in the 8K space of effectors from *Psy* and *Xcc* and *Rps* strain found in 10,000 degree-preserving simulations (grey) versus the observed number (red arrow).
