# Supplementary material for: EffectorK, a comprehensive resource to mine for Ralstonia, Xanthomonas, and other published effector interactors in the Arabidopsis proteome
Source: Mol Plant Pathol. 2020 Aug 15;21(10):1257–70. doi: 10.1111/mpp.12965 (PMC7488465; doi:10.1111/mpp.12965)
Supplement: Supplementary file 4 — FIGURE S4 Effector degree distribution for Ath effector interactors [file MPP-21-1257-s004.docx]

**A**

**B**

Published large-scale screenings

Manual curation of literature

Our large-scale screenings

Origin of data

Bacteria

Chromista

Fungi

Animalia

Origin of effectors

**Fig S4. Effector degree distribution for *Ath* effector interactors.**

Effector degree (i.e., number of effectors that interact with an *Ath* protein) distribution among the 564 identified *Ath* effector interactors (A), according to the origin the data: published large-scale screenings in light green, manual curation of literature in mid-green and this study in dark grey or (B), according to the kingdom of the corresponding effector pathogen: Bacteria in light blue, Chromista in dark blue, Fungi in light orange and Animalia in dark orange.
