# Supplementary material for: EffectorK, a comprehensive resource to mine for Ralstonia, Xanthomonas, and other published effector interactors in the Arabidopsis proteome
Source: Mol Plant Pathol. 2020 Aug 15;21(10):1257–70. doi: 10.1111/mpp.12965 (PMC7488465; doi:10.1111/mpp.12965)
Supplement: Supplementary file 5 — FIGURE S5 Ath degree and betweenness centrality of different groups of Ath effector interactors [file MPP-21-1257-s005.docx]

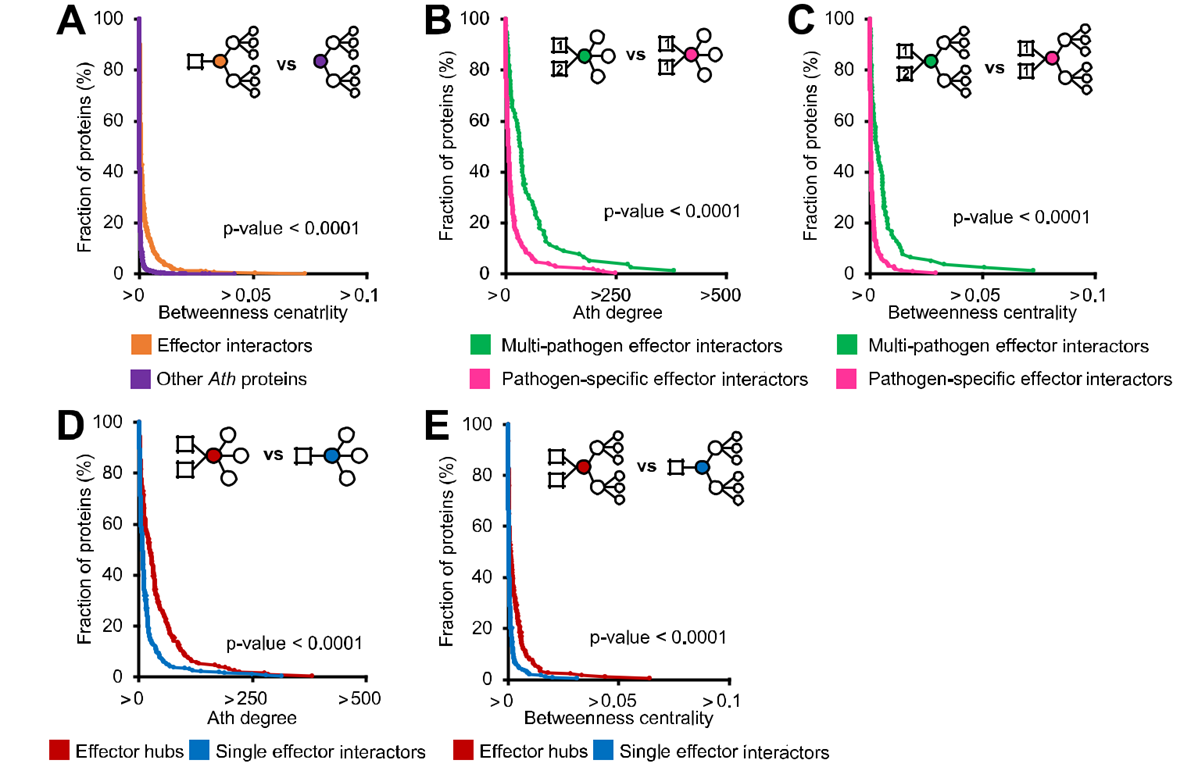


**Fig S5. *Ath* degree and betweenness centrality of different groups of *Ath* effector interactors.**

Cumulative distribution of *Ath* degree (B and D) and betweenness centrality (A, C and E) for *Ath* proteins interacting (orange) or not (purple) with effectors (B), multi-pathogen (green) and pathogen-specific (pink) effector interactors (B and C) and effector hubs (red) and single effector interactors (blue) (D and E). The significance of the differences were evaluated by one-tailed Wilcoxon signed-rank test. The illustration in the upper right corner of each graph represents each compared group: effectors are represented by squares, *Ath* proteins by circles, numbers represent different pathogens species and the color code matches the respective cumulative distribution graph. The estimation of the area under the curve of each distribution is compiled in Table 2.
