# Supplementary material for: EffectorK, a comprehensive resource to mine for Ralstonia, Xanthomonas, and other published effector interactors in the Arabidopsis proteome
Source: Mol Plant Pathol. 2020 Aug 15;21(10):1257–70. doi: 10.1111/mpp.12965 (PMC7488465; doi:10.1111/mpp.12965)
Supplement: Supplementary file 6 — FIGURE S6 Ath and effector degree of effector interactors [file MPP-21-1257-s006.docx]

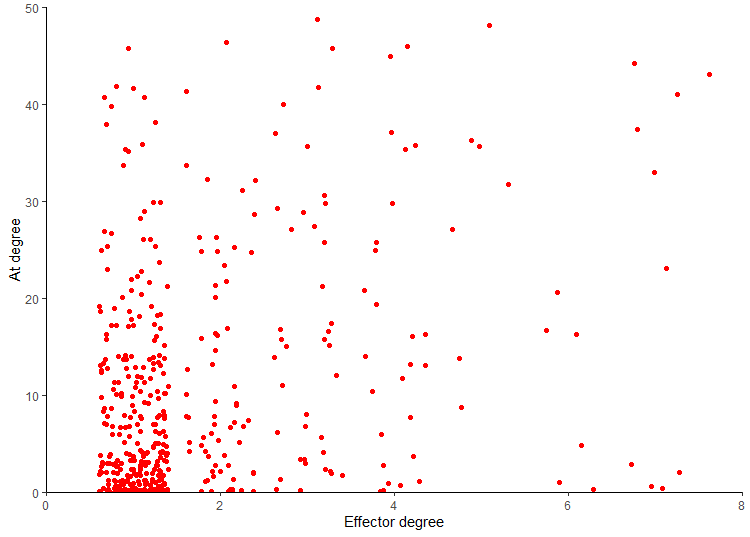


10

40

50

*Ath* degree

0

20

30

0

8

1

2

3

5

4

6

7

Effector degree


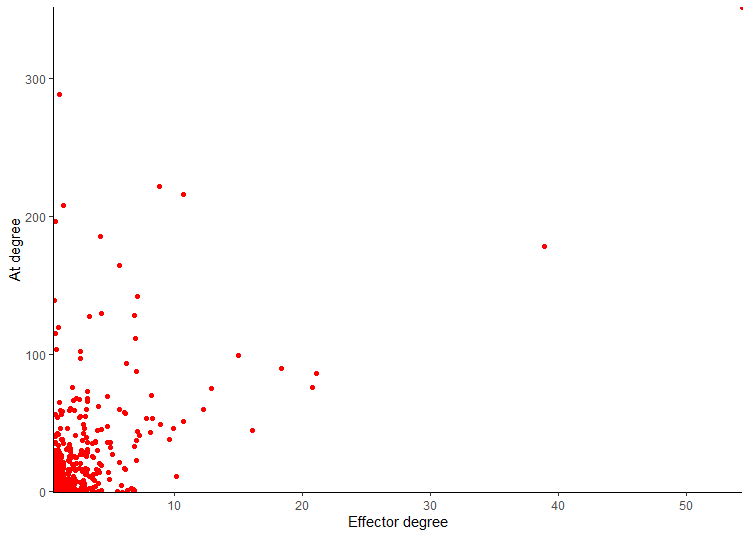


10

20

30

40

50

Effector degree

100

200

300

*Ath* degree

0

**Fig S6. *Ath* and effector degree of effector interactors.**

(A) Scatterplot of *Ath* degree versus effector degree of all *Ath* effector interactors. Squared in a grey dashed line is the close-up area represented in (B).

**A**

**B**
