# Supplementary material for: EffectorK, a comprehensive resource to mine for Ralstonia, Xanthomonas, and other published effector interactors in the Arabidopsis proteome
Source: Mol Plant Pathol. 2020 Aug 15;21(10):1257–70. doi: 10.1111/mpp.12965 (PMC7488465; doi:10.1111/mpp.12965)
Supplement: Supplementary file 7 — FIGURE S7 Degrees and betweenness centrality of bacterial core and noncore T3Es and their interactors [file MPP-21-1257-s007.docx]

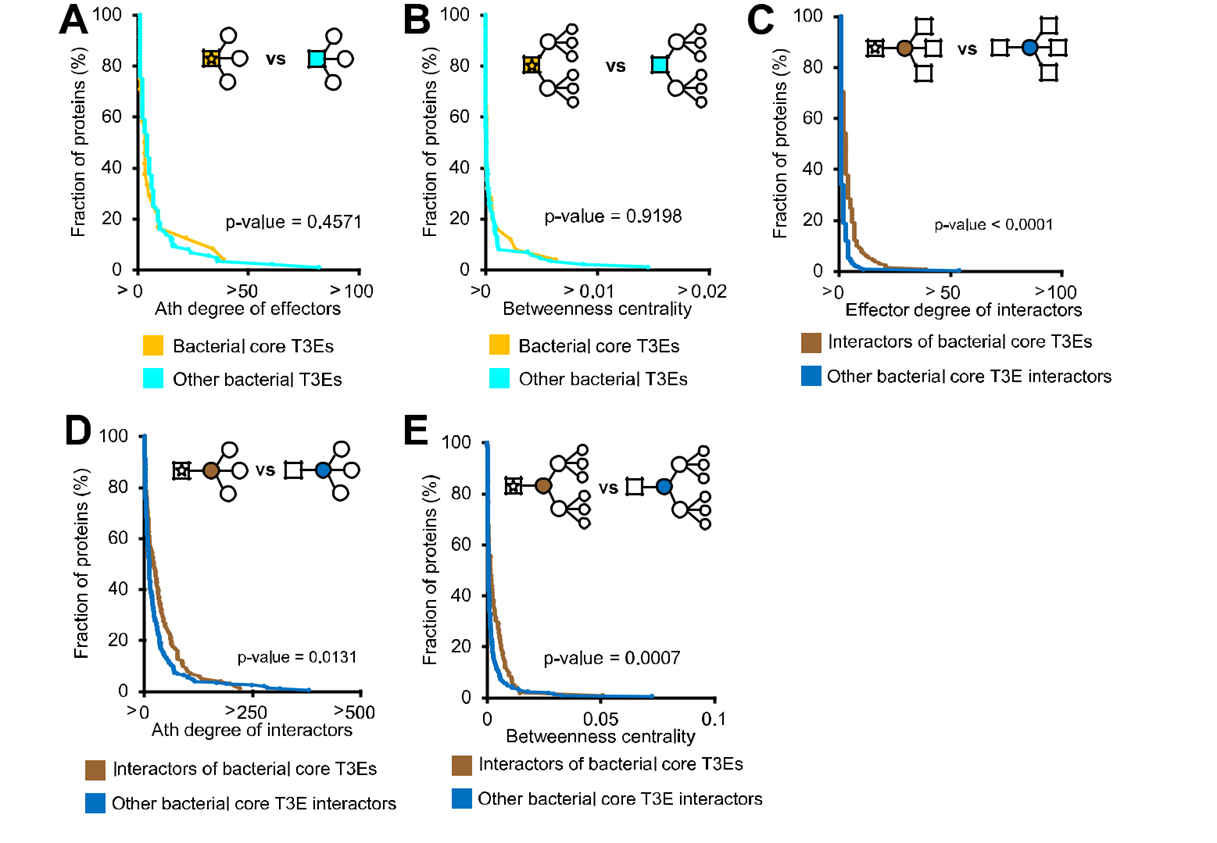


**Fig S7. Degrees and betweenness centrality of bacterial core and non-core T3Es and their interactors.**

Cumulative distribution of *Ath* degree (A and D), effector degree (C) and betweenness centrality (B and D) for bacterial core T3Es (yellow) and other bacterial T3Es (cyan) (A and B) and their interactors (brown and blue respectively) (C-E). The significance of the differences were evaluated by one-tailed Wilcoxon signed-rank test. The illustration in the upper right corner of each graph represents each compared group: bacterial T3Es are represented by squares, *Ath* proteins by circles and stars represents bacterial core T3Es. The estimation of the area under the curve of each distribution is compiled in Table 2.
